# Supplementary material for: Viroid-infected Tomato and Capsicum Seed Shipments to Australia
Source: Viruses. 2019 Jan 24;11(2):98. doi: 10.3390/v11020098 (PMC6410188; doi:10.3390/v11020098)
Supplement: Supplementary file 1 [file viruses-11-00098-s001.zip › Supplementary file 1 Viruses 415665.pdf]

Supplementary file 1. Matches to sequences in the Genbank database.

This supplementary file provides a number of the sequences obtained from seed lots and an analysis of likely associations of the isolates. Generally the longest available sequences were selected for this file and analysis.

Methods

The GenBank non-redundant nucleotide sequence database was searched for matches to the sequences by using the BLASTN (BLAST nucleotide) program. Dendrograms generated by the minimum evolution method, as implemented in BLASTN suite of programs, were used along with the BLASTN search results to find the closest matching sequences in the database and to identify clusters of isolates to which the seed isolates were likely to belong. Alignments were made using the MAFFT programs and the FFT-NS-i method, with a gap opening parameter of 1.

Isolates from seed lots were named using the following convention: viroid species, host, location, submission reference number and first initial of testing laboratory.

Host: Ca= Capsicum; To = Tomato,

Region: Eu = Europe; EA – east Asia; NK = region not known; ME = middle east; As = Asia; SAm = South America; SAs – South Asia

Lab: C=CHS; E = EAMI

References to methods

- Altschul, G.F.; Gish, W.; Miller, W.; Myers, E.W.; Lipman, D.J. Basic local alignment search tool. *J. Mol. Biol*. **1990**, *215*, 403–410.
- blastn suite. National Center for Biotechnology Information. Available online: https://blast.ncbi.nlm.nih.gov/Blast.cgi (accessed on 30 November 2018).
- Katoh, K.; Standley, D.M. MAFFT Multiple Sequence Alignment Software Version 7: Improvements in Performance and Usability. *Mol. Biol. Evol*. **2013**, *30*, 772–80.
- MAFFT alignment implementation: https://mafft.cbrc.jp/alignment/server/

Table S1. Matches to sequences in the Genbank non-redundant database.

CLVd
CLVdCaEA1302154C, CLVdToME1155E, CLVdToME1273E are closest to the sequences of a cluster of isolates that includes Rutgers 3 (JF742635.1), Prayong-16 (KC143294.1) and Chaipayong-16 (KM214217.1); BLAST searches indicated 98-99% identity and 100% coverage. CLVdToAs1385E is closest to the sequences of a cluster of isolates that includes CM10-6c1 (JF446929.1) and NC17 (AM698094.1); BLAST searches indicated 98-99% identity and 100% coverage. CLVdToNK1285E is closest to sequences of a cluster that includes PQ-49 (DQ923058) and MC-M-11 (AM698095) with 99% identity and 100% coverage.
PSTVd
PSTVdCaSAm1302191C is closest to the sequences of a cluster of isolates that includes PSTVd SJ1PL (KC707563); BLAST searches indicated 100% identity and 100% coverage. PSTVdToSAm130212C is closest to the sequences of the cluster of isolates that includes the isolates UK (AJ583449) and Naaldwijk isolate (X17268) and appears to fall within this cluster; BLAST searches indicated 98-99% identity and 98% coverage. PSTVdToSAm130211C is closest to this same cluster of isolates, but appears to fall outside the cluster; BLAST searches indicated 96% identity and 99% coverage. PSTVdToEA130225C is closest to the sequences of isolates DN12 (KR611353) and VNIIKH-08-94B (JQ889846), with searches indicating 96% identity and 100% coverage. PSTVdToSAs27617C is closest to the sequences of the cluster isolates that includes ODN (KR611362) and WS_Bor-10-232m (JQ889848); BLAST indicated with 98% identity and 99% coverage. PSTVdToEA1291E is closest to the sequences of isolates Dasongping01 (KX159281) and 6131669 (KY936882); BLAST indicated 94% identity and 100% coverage.
PCFVd

PCFVdToNK567E, PCFVdCaEu27662C and PCFVdToAs1393E are closest to sequences of a cluster of isolates represented in the database that includes the isolates NK-12c2 (JF446898) and Somjit-20 (KM214229); BLAST searches indicated 98-99% identity and 100% coverage.
TASVd
TASVdToAs1433E and TASVdToNK1435E are closest to the sequences of a cluster of isolates that includes the isolates 5458774 (KX579067) and an unnamed Tunisian isolate(DQ144506); BLAST searches indicated 94-99% identity and 100% coverage.
TCDVd
TCDVdToAf130222C and TCDVdToAf130227C are closest to the sequences of a cluster of isolates that includes GBVdC_TCDVd02 (KU714937) and an unnamed isolate from petunia in the UK (EF582393); BLAST searches indicated up to 99% identity and 96% coverage.

Figure S1. Alignment of CLVd sequences. The alignment shows there are extensive stretches of matching sequence.

```
CLVdCaEA1302154 cggaactaaactcgtggttcctgtggttcacacctgaccctgcagccatgcaaagaaaaa
CLVdToNK1285E   cggaactaaactcgtggttcctgtggttcacacctgaccctgcagccatgcaaagaaaaa
CLVdToME1155E   cggaactaaactcgtggttcctgtggttcacacctgaccctgcggccatgcaaa-aaaaa
CLVdToME1273E   cggaactaaactcgtggttcctgtggttcacacctgaccctgcagccatgcaaa-gaaaa
CLVdToAs1385E   cggaactaaactcgtggttcctgtggttcacacctgaccctgcagccatgcaaagaaaaa
                *******************************************.********** .****

CLVdCaEA1302154 aagaacgggaggaagagcgcaaagagcggtctcaggagccccggggcaactcagaccgag
CLVdToNK1285E   aagaacgggagaaagagcgc-aagagcggtctcaggagccccggggcaactcagaccgag
CLVdToME1155E   aagaacgggaggaagagcgcaaagagcggtctcaggagccccggggcaactcagaccgag
CLVdToME1273E   aagaacgggaggaagagcgcaaagagcggtctctggagccccggggcaactcagaccgag
CLVdToAs1385E   aagaacgggaggaagagcgc-aagagcggtctcaggagccccggggcaactcagaccgag
                ***********.******** ************ **************************

CLVdCaEA1302154 cgggg--tcttgaccagtggcgagcgccctgttcagacaggagtaatcccagctgaaaca
CLVdToNK1285E   cgggg--tcttgaccagtggcgagcgccctgttcagacaggagtaatcccagctgaaaca
CLVdToME1155E   cgggg--tcttgaccagtggcgagcgccctgttcagacaggagtaatcccagctgaaaca
CLVdToME1273E   cgggg--tcttgaccagtggcgagtgccctgttcagacaggagtaatcccagctgaaaca
CLVdToAs1385E   cggggatcgcggaccgagggcggaagcctgcttcagacaggagtaatccccgctgaaaca
                *****  . . ****.. ****.. ***.  ******************* *********

CLVdCaEA1302154 gggttttcacccttcctttcttctggtttccttcctctgcttcagcggcctcgcccggag
CLVdToNK1285E   gggttttcacccttcctttcttctggtttccttcctctgcttcagcggcctcgcccggag
CLVdToME1155E   gggttttcacccttcctttcttctggtttccttcctctgcttcagcggcctcgcccggag
CLVdToME1273E   gggttttcacccttcctttcttctggtttccttcctctgcttcagcggcctcgcccggag
CLVdToAs1385E   gggttttcacccttcctttcttcgggtttccttcctctgctt--gcggcctcgcccggag
                *********************** ******************  ****************

CLVdCaEA1302154 tctt---gaccagcgcaggttctgacgcgaccggtggcatcaccgag-ttcgctcaagcc
CLVdToNK1285E   tctt---gaccagcgcaggttctgacgcgaccggtggcatcaccgagtttcgctcaaacc
CLVdToME1155E   tctt---gaccagcgcaggttctgacgcgaccggtggcatcaccgag-ttcgctcaagcc
CLVdToME1273E   tctt---gaccagcgcaggttctgacgcgaccggtggtatcaccgag-ttcgctcaagcc
CLVdToAs1385E   tcttcgaatccagcgcaggttctgacgcgaccggtggcatcaccgag--ttgctcaagcc
                ****   . ****************************.*********  *.******.**

CLVdCaEA1302154 tcaacctcctttttcttcattcta-gcttggtctccgggcgagggtgtttagcccttgga
CLVdToNK1285E   tcaatctcctttttcttcattcta-gcttggtctccgggcgagggtgtttagcccttgga
CLVdToME1155E   tccacctcctttttctcta-tcta-gcttggtctccgggcgagggtgtttagcccttgga
CLVdToME1273E   tctacctcctttttctcta-tcta-gcttggtctccgggcgagggtgtttagcccttgga
CLVdToAs1385E   tcaacctcctttttctctattctacgcttggtctccgggcgagggtgtttagcccttgga
                ** *.***********..* **** ***********************************

CLVdCaEA1302154 accgcagttggttcct
CLVdToNK1285E   accgcagttggttcct
CLVdToME1155E   accgcagttggttcct
CLVdToME1273E   accgcagttggttcct
CLVdToAs1385E   accgcagttggttcct
                ****************
```

Figure S2. Alignment of PSTVd sequences.

```
PSTVdCaSAm13021 ------GGGGAAACCTGGAGCGAACTGGCAATAAG-GCGGTGGGGAGTGCCCAGCGGCCG
PSTVdToSAm13021 ----CGTGGGAAACCTGGAGCGAACTGGCAAAAGGCGCGGTGGGGAGTCCCTCGCGGCCG
PSTVdToSAm13021 -----AGGGGAAACCTGGAGCCAACTGTCAAAAGGCGCGGTGGGGAGAGCCTCGCGGCCG
PSTVdToSAs27617 -------------------------------------CGGTGGGGAGTGCCCAGCGGCCG
PSTVdToEA1291E  ATCCCCGGGGAAACCTGGAGCGAACTGGCAATAAGGACGGTGGGGAGTGCCCAGCGGCTG
PSTVdToEA130225 ------GGGGAAACTTGGAGCGAACTGGCAATAAGGACGGTGGGGAGTGCCCAGCGGCCG
                                                     **********  **. *****.*

PSTVdCaSAm13021 ACAGGAGTAATTCCCGCCGAAACAGGGTTTTCACCCTTCCTTTCTTCGGGTGTCCTTCCT
PSTVdToSAm13021 ACAGGAGTAATTCCTGCTGAAACAGGGTTTTCACCCTTCCTTTCTTCAGGTTTCCTTCCT
PSTVdToSAm13021 ACAGGAGTAATTCCAGCTGAAACAGGGTTTTCACCCTTCCTTTCTTCAGGTTTCCTTCCT
PSTVdToSAs27617 ACAGGAGTAATTCCCGCCGAAACAGGGTTTTCACCCTTCCTTTCTTCGGGGGTCCATCCT
PSTVdToEA1291E  ACAGGAGTAATCCCCGCTGANNNNNNNNNNNNACCCTTCCTTTCTTCGGGTTTCCTTCCT
PSTVdToEA130225 ACAGGAGTAATTCCCGCCGAAACAGGGTTTTCACCCTTCCTTTTTTCGGGTGTCCTTCCT
                ***********.** **.**            ***********.***.**  *** ****

PSTVdCaSAm13021 CGCGCCCGCAGGACCACCCCTCGCCCCT-TTG-CGCTGTCGCTTCGGCTACTACCCGGTG
PSTVdToSAm13021 CGCGCCCGCAGGATCACCCCTCGCCCCC-TTG-CGCTGTCGCTTCGGCTACTACCCGGTG
PSTVdToSAm13021 CGCGCCCGCAGGATCGCCCCTCGCCCCC-TTG-CGCTGTCGCTTCGGCTACTACCCGGTG
PSTVdToSAs27617 CGCGCCCGCAGGACCACCCCTCGCCCCCTTTG-CGCTGTCGCTTCGGCTACTACCCGGTG
PSTVdToEA1291E  CGCGCCCGCAGGACCACCCCTCGCCCCCTTTG-CGCTGTCGCTTCGGCTACTACCCGGTG
PSTVdToEA130225 CGCGCCCGCAGGACCACCCCTCGCCCCCTTTGAAGCAGTCGGTTCGGGTAATACCCGGTG
                *************.*.***********. ***  ** **** ***** ** *********

PSTVdCaSAm13021 GAAACAACTGAAGCTCCCGAGAACCGCTTTTTCTCTATCTTCC-TTGCTTCGGGGCGAGG
PSTVdToSAm13021 GAAACAACTGAAGCTCCCGAGAACCGCTTTTTCTCTATCTTGC-TGGTACCGGGGCGAGG
PSTVdToSAm13021 GAAACAACTGAACCTCCCGAGAACCGCTTTTTCTATATCTTGC-TGGTACCGGGTCGAGG
PSTVdToSAs27617 GAAACAACTGAAGCTCCCGAGAACCGCTTTTTCTCTATCTTACTTTG-TTCGGGGCGAGG
PSTVdToEA1291E  GAAACAACTGAAGCTCCCGAGAACCGCTTTTTCTCTATCTTTCTTTGCTTCGGGGCGAGG
PSTVdToEA130225 GAAACAACTGAAGCTCCCGAGAACCGCTTTTTCTATATCTTACTTTG-TTCGGGGCGAGG
                ************ ********************* ****** * * *  .**** *****

PSTVdCaSAm13021 GTGTTTAGCCCTTGGAACCGCAGTTGGTTCCT----------------------------
PSTVdToSAm13021 GTGTTTAGCCCTTGGAACCGCAGTTGGTTCCT----------------------------
PSTVdToSAm13021 GTGTTTAGACCTTGGAACCACAGTTGGTTCCT----------------------------
PSTVdToSAs27617 GTGTTTAGCCCTTGGAACCGCAGTTGGTTCCTCGGAACTAAACTCGTGGTTCCTGTGGTT
PSTVdToEA1291E  GTGTTTAGCCCTTGGAACCGCAGTTGGTTCCTCGGAACTAAACTCGTGGTTCCTGTGGTT
PSTVdToEA130225 GTGTTTAGCCC-------------------------------------------------
                ******** **

PSTVdCaSAm13021 ------------------------------------------------------------
PSTVdToSAm13021 ------------------------------------------------------------
PSTVdToSAm13021 ------------------------------------------------------------
PSTVdToSAs27617 CACACCTGACCTCCTAACAATGCAAAGAAAAAAGAAGGCGGCTCGGAGGAGCGCTTCAGG
PSTVdToEA1291E  CACACCTGACCTCCTGAGCA-GAAAAGAAAAAAGAAGGCGGCTCGGAGGAGCGCTTCAGG
PSTVdToEA130225 ------------------------------------------------------------


PSTVdCaSAm13021 -
PSTVdToSAm13021 -
PSTVdToSAm13021 -
PSTVdToSAs27617 A
PSTVdToEA1291E  G
PSTVdToEA130225 -
```

Figure S3. Alignment of PCFVd sequences.

```
PCFVdToNK567E;  ccggattcttctaagggtgcctgtggtgcctcccccgaagcccgcctagggaaaaagaaa
PCFVdCaEu27662C ccggattcttctaagggtgcctgtggtgcctcccccgaagcccgcttagggaaaaagaaa
PCFVdToAs1393E  ccggattcttctaagggtgcctgtggtgcctcccccgaagcccgcttagggaaaaagaaa
                *********************************************.**************

PCFVdToNK567E;  ggggaagcaagcatctcctgttcagggatccccggggaaacctggacagaccgggcggag
PCFVdCaEu27662C ggggaagcaagcatctcctgttcagggatccccggggaaacctggacagaccgggcggag
PCFVdToAs1393E  ggggaagcaagcatctcctgttcagggatccccggggaaacctgaacagatcgggcggaa
                ********************************************.*****.********.

PCFVdToNK567E;  aagcgcacgagcgggaccgtcttctgacaggagtaatccccgctgaaacagggttttcac
PCFVdCaEu27662C aagcgcacgagcgggaccgtcttctgacaggagtaatccccgctgaaacagggttttcac
PCFVdToAs1393E  aagcgc-cgtgcgggtccgtcttctgacaggactannnnnnnnnnnnnnnnnnnnnnnnn
                ****** ** ***** **************** **
```

```
PCFVdToNK567E;  ccttcctttcttcgggtttccttcctcagtcgaccggtccgcgtcggccttctcgcgcac
PCFVdCaEu27662C ccttcctttcttcgggtttccttcctcagtcgaccggtccgcgtcggccttctcgcgcac
PCFVdToAs1393E  nnnnnnnnnnnnnnnnnnnnnttcctcagtcgaccggaccgcgtcggccttctcgcgcac
                                     **************** **********************

PCFVdToNK567E;  tgctgtccggctactacccggtggatacaactgacagaggtgcttttcttccacccaac
PCFVdCaEu27662C tgctgtccggctactacccggtggatacaactgacagaggtgcttttcttccacccaac
PCFVdToAs1393E  ttctgttcggagactacccggtggatacaactgacagaggtgcttttcttccaccctgc
                * ****.***  ********************************************* .*

PCFVdToNK567E;  ttctaccgacgcggccgggagtgaagctacccgggacccgagaggatct
PCFVdCaEu27662C ttctaccgacgcggccgggagtgaagctacccgggacccgagaggatct
PCFVdToAs1393E  ttctaccgacgcggccgggagtgaagctacccgggacccgagaggatct
                *************************************************
```

Figure S4. Alignment of TASVd sequences.

```
TASVdToAs1433E  catcaagaaaaaagataggcgcgggagcgaagaagtccttcagggatccccggggaaacc
TASVdToNK1435E  catcaagaaaaaagataggcgcgggagcgaagaagtccttcagggatccccggggaaacc
                ************************************************************

TASVdToAs1433E  cgaaggaagtcgaggacgggggcttccgactactcccgtgtgagacaggagtaatcccag
TASVdToNK1435E  cgaaggaagtcgaggacgggggcttccgactactcccgtgtgagacaggagtaatcccag
                ************************************************************

TASVdToAs1433E  ctgaaacagggttttcacccttcctttcttctggtttccttcctctcgcctggagaggtc
TASVdToNK1435E  ctgaaacagggttttcacccttcctttcttctggtttccttcctctcgcctggagaggtc
                ************************************************************

TASVdToAs1433E  ttcggccctcgcccggagcttctcttaggagactacccggtggatacaactgaagcttca
TASVdToNK1435E  ttcggccctcgcccggagcttctcttaggagactacccggtggatacaactgaagcttca
                ************************************************************

TASVdToAs1433E  acctccacgcgctttttctctatctttgttgctctccgggcgagggtgaaagcccgtgga
TASVdToNK1435E  acctcctcgcgctttttctctatctttgttgctctccgggcgagggtgaaagcccgtgga
                ****** *****************************************************

TASVdToAs1433E  accctactaagagtccct
TASVdToNK1435E  accctagtaagagtccct
                ****** ***********
```

Figure S5. Alignment of TCDVd sequences. The sequences are identical over most of their length.

```
TCDVdToAf130222 g----------------------------------------------------aacagga
TCDVdToAf       ggggaaacctggacggaactggcaaaaggcggcagggagcttgtggaaggcgaaacagga
                *                                                    *******

TCDVdToAf130222 gtaatcccgagagaaacagggttttcacccttcctttctgattcggtttccttcctttgc
TCDVdToAf       gtaatcccgagagaaacagggttttcacccttcctttctgattcggtttccttcctttgc
                ************************************************************


TCDVdToAf130222 gcgccactcgacccctcgccccccttgcgctgtcgcttcggcaactacccggtggaaacaa
TCDVdToAf       gcgccactcgacccctcgccccccttgcgctgtcgcttcggcaactacccggtggaaacaa
                ************************************************************

TCDVdToAf130222 ctgaagctcccgagaaccgctttttctctatcttgctgctaccggggcgagggtgtttag
TCDVdToAf       ctgaagctcccgagaaccgctttttctctatcttgctgctaccggggcgagggtgtttag
                ************************************************************

TCDVdToAf130222 cccttggaaccgcagttggttcctcggaactaaactcgtggttcctgtggttcagacctg
TCDVdToAf       cccttggaaccgcagttggttcctcggaactaaactcgtggttcctgtggttcacacctg
                ****************************************************** *****

TCDVdToAf130222 acctcctgtgcagaaaagaaaaaagataggcggctcggaggagcgcttcagga-------
TCDVdToAf       acctcctgtgcagaaaagaaaaaagataggcggctcggaggagcgcttcaggataggcca
                *****************************************************

TCDVdToAf130222 ---
TCDVdToAf       tgt
```

Sequences
>CLVdCaEA1302154C
CGGAACTAAACTCGTGGTTCCTGTGGTTCACACCTGACCCTGCAGCCATGCAAAGAAAAAAAGAACGGGAGGAAGAGCG
CAAAGAGCGGTCTCAGGAGCCCCGGGGCAACTCAGACCGAGCGGGGTCTTGACCAGTGGCGAGCGCCCTGTTCAGACA
GGAGTAATCCCAGCTGAAACAGGGTTTTCACCCTTCCTTTCTTCTGGTTTCCTTCCTCTGCTTCAGCGGCCTCGCCCGGAGT
CTTGACCAGCGCAGGTTCTGACGCGACCGGTGGCATCACCGAGTTCGCTCAAGCCTCAACCTCCTTTTTCTTCATTCTAGCT
TGGTCTCCGGGCGAGGGTGTTTAGCCCTTGGAACCGCAGTTGGTTCCT

>CLVdToNK1285E
CGGAACTAAACTCGTGGTTCCTGTGGTTCACACCTGACCCTGCAGCCATGCAAAGAAAAAAAGAACGGGAGAAAGAGCG
CAAGAGCGGTCTCAGGAGCCCCGGGGCAACTCAGACCGAGCGGGGTCTTGACCAGTGGCGAGCGCCCTGTTCAGACAG
GAGTAATCCCAGCTGAAACAGGGTTTTCACCCTTCCTTTCTTCTGGTTTCCTTCCTCTGCTTCAGCGGCCTCGCCCGGAGTC
TTGACCAGCGCAGGTTCTGACGCGACCGGTGGCATCACCGAGTTTCGCTCAAACCTCAATCTCCTTTTTCTTCATTCTAGCT
TGGTCTCCGGGCGAGGGTGTTTAGCCCTTGGAACCGCAGTTGGTTCCT

>CLVdToME1155E
CGGAACTAAACTCGTGGTTCCTGTGGTTCACACCTGACCCTGCGGCCATGCAAAAAAAAAAGAACGGGAGGAAGAGCGC
AAAGAGCGGTCTCAGGAGCCCCGGGGCAACTCAGACCGAGCGGGGTCTTGACCAGTGGCGAGCGCCCTGTTCAGACAG
GAGTAATCCCAGCTGAAACAGGGTTTTCACCCTTCCTTTCTTCTGGTTTCCTTCCTCTGCTTCAGCGGCCTCGCCCGGAGTC
TTGACCAGCGCAGGTTCTGACGCGACCGGTGGCATCACCGAGTTCGCTCAAGCCTCCACCTCCTTTTTCTCTATCTAGCTTG
GTCTCCGGGCGAGGGTGTTTAGCCCTTGGAACCGCAGTTGGTTCCT

>CLVdToME1273E
CGGAACTAAACTCGTGGTTCCTGTGGTTCACACCTGACCCTGCAGCCATGCAAAGAAAAAAGAACGGGAGGAAGAGCGC
AAAGAGCGGTCTCTGGAGCCCCGGGGCAACTCAGACCGAGCGGGGTCTTGACCAGTGGCGAGTGCCCTGTTCAGACAG
GAGTAATCCCAGCTGAAACAGGGTTTTCACCCTTCCTTTCTTCTGGTTTCCTTCCTCTGCTTCAGCGGCCTCGCCCGGAGTC
TTGACCAGCGCAGGTTCTGACGCGACCGGTGGTATCACCGAGTTCGCTCAAGCCTCTACCTCCTTTTTCTCTATCTAGCTTG
GTCTCCGGGCGAGGGTGTTTAGCCCTTGGAACCGCAGTTGGTTCCT

>CLVdToAs1385E
CGGAACTAAACTCGTGGTTCCTGTGGTTCACACCTGACCCTGCAGCCATGCAAAGAAAAAAAGAACGGGAGGAAGAGCG
CAAGAGCGGTCTCAGGAGCCCCGGGGCAACTCAGACCGAGCGGGGATCGCGGACCGAGGGCGGAAGCCTGCTTCAGAC
AGGAGTAATCCCCGCTGAAACAGGGTTTTCACCCTTCCTTTCTTCGGGTTTCCTTCCTCTGCTTGCGGCCTCGCCCGGAGTC
TTCGAATCCAGCGCAGGTTCTGACGCGACCGGTGGCATCACCGAGTTGCTCAAGCCTCAACCTCCTTTTTCTCTATTCTACG
CTTGGTCTCCGGGCGAGGGTGTTTAGCCCTTGGAACCGCAGTTGGTTCCT

>PSTVdCaSAm1302191C
GGGGAAACCTGGAGCGAACTGGCAATAAGGCGGTGGGGAGTGCCCAGCGGCCGACAGGAGTAATTCCCGCCGAAACAG
GGTTTTCACCCTTCCTTTCTTCGGGTGTCCTTCCTCGCGCCCGCAGGACCACCCCTCGCCCCTTTGCGCTGTCGCTTCGGCTA
CTACCCGGTGGAAACAACTGAAGCTCCCGAGAACCGCTTTTTCTCTATCTTCCTTGCTTCGGGGCGAGGGTGTTTAGCCCT
TGGAACCGCAGTTGGTTCCT

>PSTVdToSAm130212C
CGTGGGAAACCTGGAGCGAACTGGCAAAAGGCGCGGTGGGGAGTCCCTCGCGGCCGACAGGAGTAATTCCTGCTGAAA
CAGGGTTTTCACCCTTCCTTTCTTCAGGTTTCCTTCCTCGCGCCCGCAGGATCACCCCTCGCCCCCTTGCGCTGTCGCTTCGG
CTACTACCCGGTGGAAACAACTGAAGCTCCCGAGAACCGCTTTTTCTCTATCTTGCTGGTACCGGGGCGAGGGTGTTTAGC
CCTTGGAACCGCAGTTGGTTCCT

>PSTVdToSAm130211C
AGGGGAAACCTGGAGCCAACTGTCAAAAGGCGCGGTGGGGAGAGCCTCGCGGCCGACAGGAGTAATTCCAGCTGAAAC
AGGGTTTTCACCCTTCCTTTCTTCAGGTTTCCTTCCTCGCGCCCGCAGGATCGCCCCTCGCCCCCTTGCGCTGTCGCTTCGGC
TACTACCCGGTGGAAACAACTGAACCTCCCGAGAACCGCTTTTTCTATATCTTGCTGGTACCGGGTCGAGGGTGTTTAGAC
CTTGGAACCACAGTTGGTTCCT

>PSTVdToEA130225C
GGGGAAACTTGGAGCGAACTGGCAATAAGGACGGTGGGGAGTGCCCAGCGGCCGACAGGAGTAATTCCCGCCGAAACA
GGGTTTTCACCCTTCCTTTTTTCGGGTGTCCTTCCTCGCGCCCGCAGGACCACCCCTCGCCCCCTTTGAAGCAGTCGGTTCG
GGTAATACCCGGTGGAAACAACTGAAGCTCCCGAGAACCGCTTTTTCTATATCTTACTTTGTTCGGGGCGAGGGTGTTTAG
CCC

>PSTVdToSAs27617C
CGGTGGGGAGTGCCCAGCGGCCGACAGGAGTAATTCCCGCCGAAACAGGGTTTTCACCCTTCCTTTCTTCGGGGGTCCAT
CCTCGCGCCCGCAGGACCACCCCTCGCCCCCTTTGCGCTGTCGCTTCGGCTACTACCCGGTGGAAACAACTGAAGCTCCCG
AGAACCGCTTTTTCTCTATCTTACTTTGTTCGGGGCGAGGGTGTTTAGCCCTTGGAACCGCAGTTGGTTCCTCGGAACTAA
ACTCGTGGTTCCTGTGGTTCACACCTGACCTCCTAACAATGCAAAGAAAAAAGAAGGCGGCTCGGAGGAGCGCTTCAGG
A

>PSTVdToEA1291E
ATCCCCGGGGAAACCTGGAGCGAACTGGCAATAAGGACGGTGGGGAGTGCCCAGCGGCTGACAGGAGTAATCCCCGCT
GAnnnnnnnnnnnnACCCTTCCTTTCTTCGGGTTTCCTTCCTCGCGCCCGCAGGACCACCCCTCGCCCCCTTTGCGCTGTCGC
TTCGGCTACTACCCGGTGGAAACAACTGAAGCTCCCGAGAACCGCTTTTTCTCTATCTTTCTTTGCTTCGGGGCGAGGGTG
TTTAGCCCTTGGAACCGCAGTTGGTTCCTCGGAACTAAACTCGTGGTTCCTGTGGTTCACACCTGACCTCCTGAGCAGAAA
AGAAAAAAGAAGGCGGCTCGGAGGAGCGCTTCAGGG

> PCFVdToNK567E; this sequence is available in GenBank as KC762952.1
CCGGATTCTTCTAAGGGTGCCTGTGGTGCCTCCCCCGAAGCCCGCCTAGGGAAAAAGAAAGGGGAAGCAAGCATCTCCT
GTTCAGGGATCCCCGGGGAAACCTGGACAGACCGGGCGGAGAAGCGCACGAGCGGGACCGTCTTCTGACAGGAGTAAT
CCCCGCTGAAACAGGGTTTTCACCCTTCCTTTCTTCGGGTTTCCTTCCTCAGTCGACCGGTCCGCGTCGGCCTTCTCGCGCA
CTGCTGTCCGGCTACTACCCGGTGGATACAACTGACAGAGGTGCTTTTTCTTCCACCCAACTTCTACCGACGCGGCCGGGA
GTGAAGCTACCCGGGACCCGAGAGGATCT

>PCFVdCaEu27662C
CCGGATTCTTCTAAGGGTGCCTGTGGTGCCTCCCCCGAAGCCCGCTTAGGGAAAAAGAAAGGGGAAGCAAGCATCTCCT
GTTCAGGGATCCCCGGGGAAACCTGGACAGACCGGGCGGAGAAGCGCACGAGCGGGACCGTCTTCTGACAGGAGTAAT
CCCCGCTGAAACAGGGTTTTCACCCTTCCTTTCTTCGGGTTTCCTTCCTCAGTCGACCGGTCCGCGTCGGCCTTCTCGCGCA
CTGCTGTCCGGCTACTACCCGGTGGATACAACTGACAGAGGTGCTTTTTCTTCCACCCAACTTCTACCGACGCGGCCGGGA
GTGAAGCTACCCGGGACCCGAGAGGATCT

>PCFVdToAs1393E
CCGGATTCTTCTAAGGGTGCCTGTGGTGCCTCCCCCGAAGCCCGCTTAGGGAAAAAGAAAGGGGAAGCAAGCATCTCCT
GTTCAGGGATCCCCGGGGAAACCTGAACAGATCGGGCGGAAAAGCGCCGTGCGGGTCCGTCTTCTGACAGGACTANNN
NNNNNNNNNNNNNNNNNNNNNNNNNNNNNNNNNNNNNNTTCCTCAGTCGACCGGACCGCGTCGGCCTTC
TCGCGCACTTCTGTTCGGAGACTACCCGGTGGATACAACTGACAGAGGTGCTTTTTCTTCCACCCTGCTTCTACCGACGCG
GCCGGGAGTGAAGCTACCCGGGACCCGAGAGGATCT

>TASVdToAs1433E
CATCAAGAAAAAAGATAGGCGCGGGAGCGAAGAAGTCCTTCAGGGATCCCCGGGGAAACCCGAAGGAAGTCGAGGAC
GGGGGCTTCCGACTACTCCCGTGTGAGACAGGAGTAATCCCAGCTGAAACAGGGTTTTCACCCTTCCTTTCTTCTGGTTTC
CTTCCTCTCGCCTGGAGAGGTCTTCGGCCCTCGCCCGGAGCTTCTCTTAGGAGACTACCCGGTGGATACAACTGAAGCTTC
AACCTCCACGCGCTTTTTCTCTATCTTTGTTGCTCTCCGGGCGAGGGTGAAAGCCCGTGGAACCCTACTAAGAGTCCCT

>TASVdToNK1435E
CATCAAGAAAAAAGATAGGCGCGGGAGCGAAGAAGTCCTTCAGGGATCCCCGGGGAAACCCGAAGGAAGTCGAGGAC
GGGGGCTTCCGACTACTCCCGTGTGAGACAGGAGTAATCCCAGCTGAAACAGGGTTTTCACCCTTCCTTTCTTCTGGTTTC
CTTCCTCTCGCCTGGAGAGGTCTTCGGCCCTCGCCCGGAGCTTCTCTTAGGAGACTACCCGGTGGATACAACTGAAGCTTC
AACCTCCTCGCGCTTTTTCTCTATCTTTGTTGCTCTCCGGGCGAGGGTGAAAGCCCGTGGAACCCTAGTAAGAGTCCCT

>TCDVdToAf130222C
GAACAGGAGTAATCCCGAGAGAAACAGGGTTTTCACCCTTCCTTTCTGATTCGGTTTCCTTCCTTTGCGCGCCACTCGACCC
CTCGCCCCCTTGCGCTGTCGCTTCGGCAACTACCCGGTGGAAACAACTGAAGCTCCCGAGAACCGCTTTTTCTCTATCTTGC

TGCTACCGGGGCGAGGGTGTTTAGCCCTTGGAACCGCAGTTGGTTCCTCGGAACTAAACTCGTGGTTCCTGTGGTTCAGA
CCTGACCTCCTGTGCAGAAAAGAAAAAAGATAGGCGGCTCGGAGGAGCGCTTCAGGA

> TCDVdToAf130227C
GGGGAAACCTGGACGGAACTGGCAAAAGGCGGCAGGGAGCTTGTGGAAGGCGAAACAGGAGTAATCCCGAGAGAAAC
AGGGTTTTCACCCTTCCTTTCTGATTCGGTTTCCTTCCTTTGCGCGCCACTCGACCCCTCGCCCCCTTGCGCTGTCGCTTCGG
CAACTACCCGGTGGAAACAACTGAAGCTCCCGAGAACCGCTTTTTCTCTATCTTGCTGCTACCGGGGCGAGGGTGTTTAGC
CCTTGGAACCGCAGTTGGTTCCTCGGAACTAAACTCGTGGTTCCTGTGGTTCACACCTGACCTCCTGTGCAGAAAAGAAAA
AAGATAGGCGGCTCGGAGGAGCGCTTCAGGATAGGCCATGT
