# Supplementary material for: Viroid-infected Tomato and Capsicum Seed Shipments to Australia
Source: Viruses. 2019 Jan 24;11(2):98. doi: 10.3390/v11020098 (PMC6410188; doi:10.3390/v11020098)
Supplement: Supplementary file 1 [file viruses-11-00098-s001.zip › Supplementary file 2 Viruses 415665.pdf]

Supplementary file 2. Isolates with identical sequences obtained from different seed lots.
This supplementary file provides alignments of viroid sequences obtained from different seed lots which were identified as being 100% identical to each other or nearly identical.

Methods
All sequences of the same viroid species were aligned and compared using MEGA7 to identify identical sequences. Sequences of any length were selected as long as they were of sufficient quality for analysis. Multiple alignments were made using the MUSCLE program running with the default parameters. The evolutionary distances were computed in MEGA7 using default parameters. To confirm and visualise the extent of the sequence similarity, identical isolates were re-aligned using CLUSTAL OMEGA. The GenBank non-redundant nucleotide sequence database was searched for matches to the sequences by using the blastn (BLAST nucleotide) program. All the BLASTN matches reported in this document covered 100% of the query sequences, excluding any unresolved nucleotide positions ('N').

References

- Altschul, G.F.; Gish, W.; Miller, W.; Myers, E.W.; Lipman, D.J. Basic local alignment search tool. *J. Mol. Biol*. **1990**, *215*, 403–410.
- Edgar, R.C. MUSCLE: multiple sequence alignment with high accuracy and high throughput. *Nucleic Acids Res.* 2004, 32, 1792–1797.
- Katoh, K.; Standley, D.M. MAFFT Multiple Sequence Alignment Software Version 7: Improvements in Performance and Usability. Mol. Biol. Evol. 2013, 30, 772–80.
- Li, W.; Cowley, A.; Uludag, M.; Gur, T.; McWilliam, H.; Squizzato, S.; Park, Y.M.; Buso, B.; Lopez, R. The EMBL-EBI bioinformatics web and programmatic tools framework. *Nucleic Acids Res.* **2015**, *43*, W580–W584, doi:10.1093/nar/gkv279.
- Sievers, F.; Wilm, A.; Dineen, D.; Gibson, T. J.; Karplus, K.; Li, W.; Lopez, R.; McWilliam, H.; Remmert, M.; Söding, J.; Thompson, J. D.; Higgins, D.G. Fast, scalable generation of high-quality protein multiple sequence alignments using Clustal Omega. Mol. Syst. Biol. 2011, 7, 539. doi:10.1038/msb.2011.75
- Blast searches of GenBank: https://blast.ncbi.nlm.nih.gov/Blast.cgi
- CLUSTAL OMEGA: https://www.ebi.ac.uk/Tools/msa/clustalo/
- MAFFT alignments: https://mafft.cbrc.jp/alignment/server/

Isolates from seed lots were named using the following convention: viroid species, host, location, submission reference number and first initial of testing laboratory:
Host: Ca= Capsicum; To = Tomato,
Region: Af = Africa; As = Asia; Eu = Europe; EA – east Asia; ME = middle east; NAm = North America; NK = region not known; SAm = South America; SAs – South Asia
Lab: C=CHS; E = EAMI

Figure S6. Identical PSTVd sequences obtained from different seed lots. PSTVdToNK12/744E, PSTVdCaNam14/675E, PSTVdCaNAm14/627E and PSTVdCaNAm15/1046E are identical; they have 100% nucleotide identity to the Chinese Potato spindle tuber viroid isolate ODN (KR611362) and others.

```
PSTVdToNK12/744E        CTGGAGCGAACTGGCAATAAGGACGGTGGGGAGTGCCCAGCGGCCGACAGGAGTAATTCC  60
PSTVdCaNam14/675E       CTGGAGCGAACTGGCAATAAGGACGGTGGGGAGTGCCCAGCGGCCGACAGGAGTAATTCC  60
PSTVdCaNAm14/627E       CTGGAGCGAACTGGCAATAAGGACGGTGGGGAGTGCCCAGCGGCCGACAGGAGTAATTCC  60
PSTVdCaNAm15/1046E      CTGGAGCGAACTGGCAATAAGGACGGTGGGGAGTGCCCAGCGGCCGACAGGAGTAATTCC  60
                        ************************************************************

PSTVdToNK12/744E        CGCCGAAACAGGGTTTTCACCCTTCCTTTCTTCGGGTGTCCTTCCTCGCGCCCGCAGGTC  120
PSTVdCaNam14/675E       CGCCGAAACAGGGTTTTCACCCTTCCTTTCTTCGGGTGTCCTTCCTCGCGCCCGCAGGTC  120
PSTVdCaNAm14/627E       CGCCGAAACAGGGTTTTCACCCTTCCTTTCTTCGGGTGTCCTTCCTCGCGCCCGCAGGTC  120
PSTVdCaNAm15/1046E      CGCCGAAACAGGGTTTTCACCCTTCCTTTCTTCGGGTGTCCTTCCTCGCGCCCGCAGGTC  120
                        ************************************************************

PSTVdToNK12/744E        CACCCCTCGCCCCCTTTGCGCTGTCGCTTCGGCTAC-      156
PSTVdCaNam14/675E       CACCCCTCGCCCCCTTTGCGCTGTCGCTTCGGCTACT      157
PSTVdCaNAm14/627E       CACCCCTCGCCCCCTTTGCGCTGTCGCTTCGGCTACT      157
PSTVdCaNAm15/1046E      CACCCCTCGCCCCCTTTGCGCTGTCGCTTCGGCTACT      157
                        ************************************
```

Figure S7. PSTVd sequences obtained from different seed lots. PSTVdToNK13/567E, PSTVdCaEu13/102E, PSTVdToNK12/868E and PSTVdToNK13/020E are identical and were found to have 98% nucleotide identity to the Polish experimental isolate Potato spindle tuber viroid isolate S7 (AY492081).

```
PSTVdToEu13/101bE      ----------------------------GGGTAGTGCCCAGCGGCCGACAGGAGTAATTC       32
PSTVdToNK12/868E       CTGGAGTGAACTGGCAAAAAAGGACGGTGGGGAGTGCCCAGCGGCCGACAGGAGTAATTC       60
PSTVdToNK13/567E       CTGGAGCGAACTGGCAAAAAAGGACGGTGGGGAGTGCCCAGCGGCCGACAGGAGTAATTC       60
PSTVdCaEu13/102E       CTGGAGCGAACTGGCAAAAAAGGACGGTGGGGAGTGCCCAGCGGCCGACAGGAGTAATTC       60
PSTVdToNK13/020E       CTGGAGCGAACTGGCAAAAAAGGACGGTGGGGAGTGCCCAGCGGCCGACAGGAGTAATTC       60
                                                   *** ****************************

PSTVdToEu13/101bE      CTGCTGAAACAGGGTTTTCACCCTTCCTTTCTTCAGGTTTCCTTCCTCGCGCCCGCAGGT       92
PSTVdToNK12/868E       CTGCTGAAACAGGGTTTTCACCCTTCCTTTCTTCAGGTTTCCTTCCTCGCGCCCGCAGGT       120
PSTVdToNK13/567E       CTGCTGAAACAGGGTTTTCACCCTTCCTTTCTTCAGGTTTCCTTCCTCGCGCCCGCAGGT       120
PSTVdCaEu13/102E       CTGCTGAAACAGGGTTTTCACCCTTCCTTTCTTCAGGTTTCCTTCCTCGCGCCCGCAGGT       120
PSTVdToNK13/020E       CTGCTGAAACAGGGTTTTCACCCTTCCTTTCTTCAGGTTTCCTTCCTCGCGCCCGCAGGT       120
                       ************************************************************

PSTVdToEu13/101bE      CCACCCCTCGCCCCCTTTGCGCTGTCGCTTCGGCTACTACCCGGTGGATACAACTGAAGC       152
PSTVdToNK12/868E       CCACCCCTCGCCCCCTTTGCGCTGTCGCTTCGGCTACT----------------------       158
PSTVdToNK13/567E       CCACCCCTCGCCCCCTTTGCGCTGTCGCTTCGGCTACT----------------------       158
PSTVdCaEu13/102E       CCACCCCTCGCCCCCTTTGCGCTGTCGCTTCGGCTACT----------------------       158
PSTVdToNK13/020E       CCACCCCTCGCCCCCTTTGCGCTGTCGCTTCGGCTACT----------------------       158
                       **************************************

PSTVdToEu13/101bE      T      153
PSTVdToNK12/868E       -      158
PSTVdToNK13/567E       -      158
PSTVdCaEu13/102E       -      158
PSTVdToNK13/020E       -      158
```

Figure S8. Identical and nearly identical PSTVd sequences obtained from different seed lots. PSTVdToNK1713172C, PSTVdCaNAm14/836E and PSTVdCaAf13/124E are identical and were found to have 100% nucleotide identity to the Chinese Potato spindle tuber viroid isolate ODN (KR611362.1) and others.

```
PSTVdTo1713172C        --------------CTGGAGCGAACTGGCAATAAGGACGGTGGGGAGTGCCCAGCGGCCG  46
PSTVdCaNAm14/836E      --------------CTGGAGCGAACTGGCAATAAGGACGGTGGGGAGTGCCCAGCGGCCG  46
PSTVdCaAf13/124E       --------------CTGGAGCGAACTGGCAATAAGGACGGTGGGGAGTGCCCAGCGGCCG  46
PSTVdToME27642C        --------------CTGGAGCGAACTGGCAACAAGGACGGTGGGGAGTGCCCAGCGGCCG  46
PSTVdCaSAm1302191C     ------GGGGAAACCTGGAGCGAACTGGCAATAAG-GCGGTGGGGAGTGCCCAGCGGCCG  53
PSTVdToEAs13/161E      ATCCCCGGGGAAACCTGGAGCGAACTGGCAATAAGGACGGTGGGGAGTGCCCAGCGGCCG  60
                                     ***************** *** .***********************

PSTVdToNK1713172C      ACAGGAGTAATTCCCGCCGAAACAGGGTTTTCACCCTTCCTTTCTTCGGGTGTCCTTCCT  106
PSTVdCaNAm14/836E      ACAGGAGTAATTCCCGCCGAAACAGGGTTTTCACCCTTCCTTTCTTCGGGTGTCCTTCCT  106
PSTVdCaAf13/124E       ACAGGAGTAATTCCCGCCGAAACAGGGTTTTCACCCTTCCTTTCTTCGGGTGTCCTTCCT  106
PSTVdToME27642C        ACAGGAGTAATTCCCGCCGAAACAGGGTTTTCACCCTTCCTTTCTTCGGGTGTCCTTCCT  106
PSTVdCaSAm1302191C     ACAGGAGTAATTCCCGCCGAAACAGGGTTTTCACCCTTCCTTTCTTCGGGTGTCCTTCCT  113
PSTVdToEAs13/161E      ACAGGAGTAATTCCCGCCGAAACAGGGTTTTCACCCTTCCTTTCTTCGGGTGTCCTTCCT  120
                       ************************************************************

PSTVdToNK1713172C      CGCGCCCGCAGGACCACCCCTCGCCCCCTTTGCGCTGTCGCTTCGGCTACT---------  157
PSTVdCaNAm14/836E      CGCGCCCGCAGGACCACCCCTCGCCCCCTTTGCGCTGTCGCTTCGGCTACT---------  157
PSTVdCaAf13/124E       CGCGCCCGCAGGACCACCCCTCGCCCCCTTTGCGCTGTCGCTTCGGCTACT---------  157
PSTVdToME27642C        CGCGCCCGCAGGACCACCCCTCGCCCCCTTTGCGCTGTCGCTTCGGTTACT---------  157
PSTVdCaSAm1302191C     CGCGCCCGCAGGACCACCCCTCGCCC-CTTTGCGCTGTCGCTTCGGCTACTACCCGGTGG  172
PSTVdToEAs13/161E      CGCGCCCGCAGGACCACCCCTCGCCCCCTTTGCGCTGTCGCTTCGGCTACTACCCGGTGG  180
                       ************************** ******************* ****

PSTVdToNK1713172C      ------------------------------------------------------------  157
PSTVdCaNAm14/836E      ------------------------------------------------------------  157
PSTVdCaAf13/124E       ------------------------------------------------------------  157
PSTVdToME27642C        ------------------------------------------------------------  157
PSTVdCaSAm1302191C     AAACAACTGAAGCTCCCGAGAACCGCTTTTTCTCTATCTT-CCTTGCTTCGGGGCGAGGG  231
PSTVdToEAs13/161E      AAACAACTGAAGCTCCCGAGAACCGCTTTTTCTCTATCTTTCTTTGCTTCGGGGCGAGGG  240


PSTVdToNK1713172C      ------------------------------------------------------------  157
PSTVdCaNAm14/836E      ------------------------------------------------------------  157
PSTVdCaAf13/124E       ------------------------------------------------------------  157
PSTVdToME27642C        ------------------------------------------------------------  157
PSTVdCaSAm1302191C     TGTTTAGCCCTTGGAACCGCAGTTGGTTCCT-----------------------------  262
PSTVdToEAs13/161E      TGTTTAGCCCTTGGAACCGCAGTTGGTTCCTCGGAACTAAACTCGTGGTTCCTGTGGTTC  300


PSTVdToNK1713172C      -----------------------------------------------------------   157
PSTVdCaNAm14/836E      -----------------------------------------------------------   157
PSTVdCaAf13/124E       -----------------------------------------------------------   157
PSTVdToME27642C        -----------------------------------------------------------   157
PSTVdCaSAm1302191C     -----------------------------------------------------------   262
PSTVdToEAs13/161E      ACACCTGACCTCCTGAGCAGAAAAGAAAAAAGAAGGCGGCTCGGAGGAGCGCTTCAGGG   359
```

Figure S9. Identical and nearly identical PSTVd isolates obtained from different seed lots. PSTVdToNK12/820E, PSTVdToNK13/1051E, PSTVdToAs13/1070E, PSTVdToNK13/1100E, PSTVdToNK13/1104E and PSTVdToNAm13/1206E are identical and were found to have 100% nucleotide identity to the Australian Potato spindle tuber viroid isolate CVNBC36 (KP454054) and others.

```
PSTVdTOEu13/967E        CTGGAGCGAACTGGCAAAAGGCGCGGTGGGGAGTGCCTCGCGGCCGACAGGAGTAATTCC  60
PSTVdToNK13/1046E       CTGGAGCGAACTGGCAAAAGGCGCGGTGGGGAGTGCCTCGCGGCCGACAGGAGTAATTCC  60
PSTVdToEu27648C         --------------CAAAAGGCGCGGTGGGAAGTGCCTCGCGGCCGACAGGAGTAATTCC  46
PSTVdToNK12/820E        CTGGAGCGAACTGGCAAAAGGCGCGGTGGGGAGTGCCTCGCGGCCGACAGGAGTAATTCC  60
PSTVdToNK13/1051E       CTGGAGCGAACTGGCAAAAGGCGCGGTGGGGAGTGCCTCGCGGCCGACAGGAGTAATTCC  60
PSTVdToAs13/1070E       CTGGAGCGAACTGGCAAAAGGCGCGGTGGGGAGTGCCTCGCGGCCGACAGGAGTAATTCC  60
PSTVdToNK13/1100E       CTGGAGCGAACTGGCAAAAGGCGCGGTGGGGAGTGCCTCGCGGCCGACAGGAGTAATTCC  60
PSTVdToNK13/1104E       CTGGAGCGAACTGGCAAAAGGCGCGGTGGGGAGTGCCTCGCGGCCGACAGGAGTAATTCC  60
PSTVdToNAm13/1206E      CTGGAGCGAACTGGCAAAAGGCGCGGTGGGGAGTGCCTCGCGGCCGACAGGAGTAATTCC  60
                                      **************** *****************************

PSTVdTOEu13/967E        TGCCGAAACAGGGTTTTCACCCTTCCTTTCTTCAGGTTTCCTTCCTCGCGCCCGCAGGAT  120
PSTVdToNK13/1046E       TGCCGAAACAGGGTTTTCACCCTTCCTTTCTTCAGGTTTCCTTCCTCGCGCCCGCAGGAT  120
PSTVdToEu27648C         TGCCGAAACAGGGTTTTCACCCTTCCTTTCTTCAGGTTTCCTTCCTCGCGCCCGCAGGAT  106
PSTVdToNK12/820E        TGCCGAAACAGGGTTTTCACCCTTCCTTTCTTCAGGTTTCCTTCCTCGCGCCCGCAGGAT  120
PSTVdToNK13/1051E       TGCCGAAACAGGGTTTTCACCCTTCCTTTCTTCAGGTTTCCTTCCTCGCGCCCGCAGGAT  120
PSTVdToAs13/1070E       TGCCGAAACAGGGTTTTCACCCTTCCTTTCTTCAGGTTTCCTTCCTCGCGCCCGCAGGAT  120
PSTVdToNK13/1100E       TGCCGAAACAGGGTTTTCACCCTTCCTTTCTTCAGGTTTCCTTCCTCGCGCCCGCAGGAT  120
PSTVdToNK13/1104E       TGCCGAAACAGGGTTTTCACCCTTCCTTTCTTCAGGTTTCCTTCCTCGCGCCCGCAGGAT  120
PSTVdToNAm13/1206E      TGCCGAAACAGGGTTTTCACCCTTCCTTTCTTCAGGTTTCCTTCCTCGCGCCCGCAGGAT  120
                        ************************************************************

PSTVdTOEu13/967E        CCACCCTCGCCCCCTTGCGCTGTCGCTTCGGCTACT          155
PSTVdToNK13/1046E       CACCCCTCGCCCCCTTGGACTGTCGCTTCGTCTACT          156
PSTVdToEu27648C         CACCCCTCGCCCCCTTGCGCTGTCGCTTCGGCTACT          142
PSTVdToNK12/820E        CACCCCTCGCCCCCTTGCGCTGTCGCTTCGGCTACT          156
PSTVdToNK13/1051E       CACCCCTCGCCCCCTTGCGCTGTCGCTTCGGCTACT          156
PSTVdToAs13/1070E       CACCCCTCGCCCCCTTGCGCTGTCGCTTCGGCTACT          156
PSTVdToNK13/1100E       CACCCCTCGCCCCCTTGCGCTGTCGCTTCGGCTACT          156
PSTVdToNK13/1104E       CACCCCTCGCCCCCTTGCGCTGTCGCTTCGGCTACT          156
PSTVdToNAm13/1206E      CACCCCTCGCCCCCTTGCGCTGTCGCTTCGGCTACT          156
                        *****************  *********** *****
```

Figure S10. Identical TCDVd sequences from different seed lots. TCDVdToAf130227C andTCDVdToMEMC27642C are identical and were found to have 100% nucleotide identity with UK isolate Tomato chlorotic dwarf viroid (EF582393).

```
TCDVdToAf               ------GGGGAAACCTGGAGCGAACTGGCAAAAGGCGGCAGGGAGCTTGTGGAAGGCGAA   54
TCDVdToMEMC27642C       ATCCCCGGGGAAACCTGGAGCGAACTGGCAAAAGGCGGCAGGGAGCTTGTGGAAGGCGAA   60
                              ******************************************************

TCDVdToAf               ACAGGAGTAATCCCGAGAGAAACAGGGTTTTCACCCTTCCTTTCTGATTCGGTTTCCTTC   114
TCDVdToMEMC27642C       ACAGGAGTAATCCCGAGAGAAACAGGGTTTTCACCCTTCCTTTCTGATTCGGTTTCCTTC   120
                        ************************************************************

TCDVdToAf               CTTTGCGCGCCACTCGACCCCTCGCCCCCTTGCGCTGTCGCTTCGGCAACTACCCGGTGG   174
TCDVdToMEMC27642C       CTTTGCGCGCCACTCGACCCCTCGCCCCCTTGCGCTGTCGCTTCGGCAACTACCCGGTGG   180
                        ************************************************************

TCDVdToAf               AAACAACTGAAGCTCCCGAGAACCGCTTTTTCTCTATCTTGCTGCTACCGGGGCGAGGGT   234
TCDVdToMEMC27642C       AAACAACTGAAGCTCCCGAGAACCGCTTTTTCTCTATCTTGCTGCTACCGGGGCGAGGGT   240
                        ************************************************************

TCDVdToAf               GTTTAGCCCTTGGAACCGCAGTTGGTTCCTCGGAACTAAACTCGTGGTTCCTGTGGTTCA   294
TCDVdToMEMC27642C       GTTTAGCCCTTGGAACCGCAGTTGGTTCCTCGGAACTAAACTCGTGGTTCCTGTGGTTCA   300
                        ************************************************************

TCDVdToAf               CACCTGACCTCCTGTGCAGAAAAGAAAAAAGATAGGCGGCTCGGAGGAGCGCTTCAGGAT   354
TCDVdToMEMC27642C       CACCTGACCTCCTGTGCAGAAAAGAAAAAAGATAGGCGGCTCGGAGGAGCGCTTCAGG--   358
                        **********************************************************

TCDVdToAf               AGGCCATGT      363
TCDVdToMEMC27642C       ---------      358
```

Figure S11. Distinct CLVd sequences obtained from different seed lots. No CLVd seed isolates were identical. CLVdCaEu16169C and CLVdCaEA1302154C had one nucleotide difference between the overlapping sequences. CLVdCaEu16169C had 98% nucleotide identity to Thailand isolate Columnea latent viroid clone Prayong-16 (KC143294). CLVdCaEA1302154C had 99% nucleotide identity to Thailand isolate Columnea latent viroid clone Prayong-16 (KC143294) and others.

```
CLVdCaEu16169C      ------------------------------------------------------------    0
CLVdCaEA1302154C    CGGAACTAAACTCGTGGTTCCTGTGGTTCACACCTGACCCTGCAGCCATGCAAAGAAAAA    60

CLVdCaEu16169C      ------------------------------------------------------------    0
CLVdCaEA1302154C    AAGAACGGGAGGAAGAGCGCAAGAGCGGTCTCAGGAGCCCCTGGGGCAACTCAGACCGAG    120

CLVdCaEu16169C      -----------------------------TTCAGACAGGAGTAATACCAGCTGAAACAGG    31
CLVdCaEA1302154C    CGGGGTCTTGACCAGTGGCGAGCGCCCTGTTCAGACAGGAGTAATCCCAGCTGAAACAGG    180
                                                 **************** **************

CLVdCaEu16169C      GTTTTCACCCTTCCTTTCTTCTGGTTTCCTTCCTCTGCTTCAGCGGCCTCGCCCGGAGTC    91
CLVdCaEA1302154C    GTTTTCACCCTTCCTTTCTTCTGGTTTCCTTCCTCTGCTTCAGCGGCCTCGCCCGGAGTC    240
                    ************************************************************

CLVdCaEu16169C      TTGACCAGCGCAGGTTCTGACGCGACCGGTGGCATCACCGAGTTCGCTCAAGCCTCAACC    151
CLVdCaEA1302154C    TTGACCAGCGCAGGTTCTGACGCGACCGGTGGCATCACCGAGTTCGCTCAAGCCTCAACC    300
                    ************************************************************

CLVdCaEu16169C      TCCTTTTTCTTCATTCTAGCTTGGTCTCCGGGCGAGGGTGTTTAGCCCTTGGAACCGCAG    211
CLVdCaEA1302154C    TCCTTTTTCTTCATTCTAGCTTGGTCTCCGGGCGAGGGTGTTTAGCCCTTGGAACCGCAG    360
                    ************************************************************

CLVdCaEu16169C      TTGGTTCCTCGGAACTAAACTCGTGGTTCCTGTGGTTCACACCTGACCCTGCAGCCATGC    271
CLVdCaEA1302154C    TTGGTTCCT---------------------------------------------------    369
                    *********

CLVdCaEu16169C      AAAGAAAAAAAGAACGGGAGGAAGAGCGCAAGAGCGGTCTCAGGAGCCCCC    322
CLVdCaEA1302154C    ---------------------------------------------------    3
```

Figure S12. Distinct CLVd sequences. CLVdToAs13/1073E and CLVdToAs13/1076E were most closely related and had four nucleotide differences between the overlapping sequences.CLVdToAs13/1073E had 99% nucleotide identity to Thailand isolate CLVd isolate CM10-6c1 (JF446929). CLVdToAs13/1076E had 98% nucleotide identity to Thailand isolate CLVd isolate CM10-6c1 (JF446929).

```
CLVdToAs13/1073E    CGGAACTAAACTCGTGGTTCCTGTGGTTCACACCTGACCCTGCAGCCATGCAAAGAAAAA    60
CLVdToAs13/1076E    CGGAACTAAACTCGTGGTTCCTGTGGTTCACACCTGACCCTGCAGCCATGCAAAGAAAAA    60
                    ************************************************************

CLVdToAs13/1073E    AAGAACGGGAGGAAGAGCGCAAGAGCGGTCTCAGGAGCCCCGGGGCAACTCAGACCGAGC    120
CLVdToAs13/1076E    AAGATTGGGAGGGAGAGCGCAAGAGCGGTCTCAGGAGCCCCGGGGCAACTCAGACCGAGC    120
                    ****  ****** ***********************************************

CLVdToAs13/1073E    GGGGATCGCGGACCGAGGGCGGAAGCCTGCTTCAGACAGGAGTAATCCCCGCTGAAACAG    180
CLVdToAs13/1076E    GGGGATCGCGGACCGAGGGCGGAAGCCTGCTTCTGACAGGAGTANNNNNNNNNNNNNNNN    180
                    ********************************* **********

CLVdToAs13/1073E    GGTTTTCACCCTTCCTTTCTTCGGGTTTCCTTCCTCTGCTTGCGGCCTCGCCCGGAGTCT    240
CLVdToAs13/1076E    NNNNNN--NNNNNNNNNNNNNNNNNNNNNNTTCCTCTGCTTGCGGCCTCGCCCGGAGTCT    238
                                                  ******************************

CLVdToAs13/1073E    TCGAATCCAGCGCAGGTTCTGACGCGACCGGTGGCATCACCGAGTTGCTCAAGCCTCAAC    300
CLVdToAs13/1076E    TCGAATCCAGCGCAGGTTCTGACGCGACCGGTGGCATCACCGAGTTGCTCAAGCCTCAAC    298
                    ************************************************************

CLVdToAs13/1073E    CTCCTTTTTCTCTATTCTACGCTTGGTCTCCGGGCGAGGGTGTTTAGCCCTTGGAACCGC    360
CLVdToAs13/1076E    CTCCTTTTTCTCTATTCTACGCTTGGTCTCCGGGCGAGGGTGTTTAGCCCTTGGAACCGC    358
                    ************************************************************

CLVdToAs13/1073E    AGTTGGTTCCT    371
CLVdToAs13/1076E    AGTTGGTTCCT    369
                    ***********
```

Figure S13. Distinct CLVd sequences. CLVdToAs13/1075E and CLVdToAs13/1074E were most closely related and had two nucleotide differences between the overlapping sequences. CLVdToAs13/1074E 98% nucleotide identity to Thailand isolate CLVd isolate CM10-6c1 (JF446929). CLVdToAs13/1075E had 99% nucleotide identity to Thailand isolate CLVd isolate CM10-6c1 (JF446929.1).

```
CLVdToAs13/1074E      CGGAACTAAACTCGTGGTTCCTGTGGTTCACACCTGACCCTGCAGCCATGCAAAGAAAAA    60
CLVdToAs13/1075E      CGGAACTAAACTCGTGGTTCCTGTGGTTCACACCTGACCCTGCAGCCATGCAAAGAAAAA    59
                      ************************************************************

CLVdToAs13/1074E      AAGATTGGGAGGAAGAGCGCAAGAGCGGTCTCAGGAGCCCCGGGGCAACTCAGACCGAGC    120
CLVdToAs13/1075E      AAGAACGGGAGGAAGAGCGCAAGAGCGGTCTCAGGAGCCCCGGGGCAACTCAGACCGAGC    119
                      ****  ******************************************************

CLVdToAs13/1074E      GGGGATCGCGGACCGAGGGCGGAAGCCTGCTTCAGACAGGAGTAATCCCCGCTGAAACAG    180
CLVdToAs13/1075E      GGGGATCGCGGACCGAGGGCGGAAGCCTGCTTCAGACAGGAGTAATCCCCGCTGAAACAG    179
                      ************************************************************

CLVdToAs13/1074E      GGTTTTCACCCTTCCTTTCTTCGGGTTTCCTTCCTCTGCTTGCGGCCTCGCCCGGAGTCT    240
CLVdToAs13/1075E      GGTTTTCACCCTTCCTTTCTTCGGGTTTCCTTCCTCTGCTTGCGGCCTCGCCCGGAGTCT    239
                      ************************************************************

CLVdToAs13/1074E      TCGAATCCAGCGCAGGTTCTGACGCGACCGGTGGCATCACCGAGTTGCTCAAGCCTCATC    300
CLVdToAs13/1075E      TCGAATCCAGCGCAGGTTCTGACGCGACCGGTGGCATCACCGAGTTGCTCAAGCCTCATC    299
                      ************************************************************

CLVdToAs13/1074E      CTCCTTTTTCTCTATTCTACGCTTGGTCTCCGGGCGAGGGTGTTTAGCCCTTGGAACCGC    360
CLVdToAs13/1075E      CTCCTTTTTCTCTATTCTACGCTTGGTCTCCGGGCGAGGGTGTTTAGCCCTTGGAACCGC    359
                      ************************************************************

CLVdToAs13/1074E      AGTTGGTTCCT     371
CLVdToAs13/1075E      AGTTGGTTCCT     370
                      ***********
```

Figure S14. Identical PCFVd sequences obtained from different seed lots. The listed PCFVd seed isolates are identical to KC762952 across the available sequence. KC762952 differed from KC762953 (another isolate from the seed detections) by two nucleotides. PCFVdCaME15/707E, PCFVdToME14/0008E and PCFVdToAf14/1068E isolates are identical and have 99% nucleotide identity with isolates KC762952.1 and KC762953.1 previously found in seed tested by Australia.

```
PCFVdCaNK13/1127E      CCGGATTCTTCTAAGGGTGCCTGTGGTGCCTCCCCCGAAGCCCGCTTAGGGAAAAAGAAA  60
PCFVdToNAm13/968E      CCGGATTCTTCTAAGGGTGCCTGTGGTGCCTCCCCCGAAGCCCGCTTAGGGAAAAAGAAA  60
PCFVdCaNK13/1192E      CCGGATTCTTCTAAGGGTGCCTGTGGTGCCTCCCCCGAAGCCCGCTTAGGGAAAAAGAAA  60
KC762952.1             CCGGATTCTTCTAAGGGTGCCTGTGGTGCCTCCCCCGAAGCCCGCCTAGGGAAAAAGAAA  60
KC762953.1             CCGGATTCTTCTAAGGGTGCCTGTGGTGCCTCCCCCGAAGCCCGCTTAGGGAAAAAGAAA  60
PCFVdToAf14/1068E      ------------------------------------------------------------  0
PCFVdToME14/0008E      ------------------------------------------------------------  0
PCFVdCaME15/707E       ------------------------------------------------------------  0
PCFVdToNK13/1170E      ------------------------------------------------------------  0
PCFVdToEu13/1252E      ------------------------------------------------------------  0
PCFVdCaNAm14/628E      ------------------------------------------------------------  0
PCFVdToNAm14/675E      ------------------------------------------------------------  0
PCFVdCaEAs14/847E      ------------------------------------------------------------  0
PCFVdCaEAs14/981E      ------------------------------------------------------------  0
PCFVdCaAs14/1107E      ------------------------------------------------------------  0
PCFVdCaEAs14/1154E     ------------------------------------------------------------  0


PCFVdCaNK13/1127E      GGGGAAGCAAGCATCTCCTGTTCAGGGATCCCCGGGGAAACCTGGACAGACCGGGCGGAG  120
PCFVdToNAm13/968E      GGGGAAGCAAGCATCTCCTGTTCAGGGATCCCCGGGGAAACCTGGACAGACCGGGCGGAG  120
PCFVdCaNK13/1192E      GGGGAAGCAAGCATCTCCTGTTCAGGGATCCCCGGGGAAACCTGGACAGACCGGGCGGAG  120
KC762952.1             GGGGAAGCAAGCATCTCCTGTTCAGGGATCCCCGGGGAAACCTGGACAGACCGGGCGGAG  120
KC762953.1             GGGGAAGCAAGCATCTCCTGTTCAGGGATCCCCGGGGAAACCTGGACAGACCGGGCGGAG  120
PCFVdToAf14/1068E      -----------------------------------------CTGGACAGACCGGGCGGAG  19
PCFVdToME14/0008E      -----------------------------------------CTGGACAGACCGGGCGGAG  19
PCFVdCaME15/707E       -----------------------------------------CTGGACAGACCGGGCGGAG  19
PCFVdToNK13/1170E      -----------------------------------------CTGGACAGACCGGGCGGAG  19
PCFVdToEu13/1252E      -----------------------------------------CTGGACAGACCGGGCGGAG  19
PCFVdCaNAm14/628E      -----------------------------------------CTGGACAGACCGGGCGGAG  19
```

```
PCFVdToNAm14/675E      -----------------------------------------CTGGACAGACCGGGCGGAG  19
PCFVdCaEAs14/847E      -----------------------------------------CTGGACAGACCGGGCGGAG  19
PCFVdCaEAs14/981E      -----------------------------------------CTGGACAGACCGGGCGGAG  19
PCFVdCaAs14/1107E      -----------------------------------------CTGGACAGACCGGGCGGAG  19
PCFVdCaEAs14/1154E     -----------------------------------------CTGGACAGACCGGGCGGAG  19
                                                                ******************* 

PCFVdCaNK13/1127E      AAGCGCACGAGCGGGACCGTCTTCTGACAGGAGTAATCCCCGCTGAAACAGGGATTTTTC  180
PCFVdToNAm13/968E      AAGCGCACGAGCGGGACCGTCTTCTGACAGGAGTAATCCCCGCTGAAACAGGGATTTCTC  180
PCFVdCaNK13/1192E      AAGCGCACGAGCGGGACCGTCTTCTGACAGGAGTAATCCCCGCTGAAACAGGGTTTTCAC  180
KC762952.1             AAGCGCACGAGCGGGACCGTCTTCTGACAGGAGTAATCCCCGCTGAAACAGGGTTTTCAC  180
KC762953.1             AAGCGCACGAGCGGGACCGTCTTCTGACAGGAGTAATCCCCGCTGAAACAGGGTTTTCAC  180
PCFVdToAf14/1068E      AAGCGCACGAGCGGGACCGTCTTCTGACAGGAGTAATCCCCGCTGAAACAGGGATTTCAC  79
PCFVdToME14/0008E      AAGCGCACGAGCGGGACCGTCTTCTGACAGGAGTAATCCCCGCTGAAACAGGGATTTCTC  79
PCFVdCaME15/707E       AAGCGCACGAGCGGGACCGTCTTCTGACAGGAGTAATCCCCGCTGAAACAGGGATTTCTC  79
PCFVdToNK13/1170E      AAGCGCACGAGCGGGACCGTCTTCTGACAGGAGTAATCCCCGCTGAAACAGGGTTTTCAC  79
PCFVdToEu13/1252E      AAGCGCACGAGCGGGACCGTCTTCTGACAGGAGTAATCCCCGCTGAAACAGGGTTTTCAC  79
PCFVdCaNAm14/628E      AAGCGCACGAGCGGGACCGTCTTCTGACAGGAGTAATCCCCGCTGAAACAGGGTTTTCAC  79
PCFVdToNAm14/675E      AAGCGCACGAGCGGGACCGTCTTCTGACAGGAGTAATCCCCGCTGAAACAGGGTTTTCAC  79
PCFVdCaEAs14/847E      AAGCGCACGAGCGGGACCGTCTTCTGACAGGAGTAATCCCCGCTGAAACAGGGTTTTCAC  79
PCFVdCaEAs14/981E      AAGCGCACGAGCGGGACCGTCTTCTGACAGGAGTAATCCCCGCTGAAACAGGGTTTTCAC  79
PCFVdCaAs14/1107E      AAGCGCACGAGCGGGACCGTCTTCTGACAGGAGTAATCCCCGCTGAAACAGGGTTTTCAC  79
PCFVdCaEAs14/1154E     AAGCGCACGAGCGGGACCGTCTTCTGACAGGAGTAATCCCCGCTGAAACAGGGTTTTCAC  79
                       *****************************************************:*** :*

PCFVdCaNK13/1127E      CCTTCCTTTCTTCGGGTTTCCTTCCTCAGTCGACCGGTCCGCGTCGGCCTTCTCGCGCAC  240
PCFVdToNAm13/968E      CCTTCCTTTCTTCGGGTTTCCTTCCTCAGTCGACCGGTCCGCGTCGGCCTTCTCGCGCAC  240
PCFVdCaNK13/1192E      CCTTCCTTTCTTCGGGTTTCCTTCCTCAGTCGACCGGTCCGCGTCGGCCTTCTCGCGCAC  240
KC762952.1             CCTTCCTTTCTTCGGGTTTCCTTCCTCAGTCGACCGGTCCGCGTCGGCCTTCTCGCGCAC  240
KC762953.1             CCTTCCTTTCTTCGGGTTTCCTTCCTCAGTCGACCGGTCCGCGTCGGCCTTCTCGCGCAC  240
PCFVdToAf14/1068E      CCTTCCTTTCTTCGGGTTTCCTTCCTCAGTCGACCGGTCCGCGTCGGCCTTCTCGCGCAC  139
PCFVdToME14/0008E      CCTTCCTTTCTTCGGGTTTCCTTCCTCAGTCGACCGGTCCGCGTCGGCCTTCTCGCGCAC  139
PCFVdCaME15/707E       CCTTCCTTTCTTCGGGTTTCCTTCCTCAGTCGACCGGTCCGCGTCGGCCTTCTCGCGCAC  139
PCFVdToNK13/1170E      CCTTCCTTTCTTCGGGTTTCCTTCCTCAGTCGACCGGTCCGCGTCGGCCTTCTCGCGCAC  139
PCFVdToEu13/1252E      CCTTCCTTTCTTCGGGTTTCCTTCCTCAGTCGACCGGTCCGCGTCGGCCTTCTCGCGCAC  139
PCFVdCaNAm14/628E      CCTTCCTTTCTTCGGGTTTCCTTCCTCAGTCGACCGGTCCGCGTCGGCCTTCTCGCGCAC  139
PCFVdToNAm14/675E      CCTTCCTTTCTTCGGGTTTCCTTCCTCAGTCGACCGGTCCGCGTCGGCCTTCTCGCGCAC  139
PCFVdCaEAs14/847E      CCTTCCTTTCTTCGGGTTTCCTTCCTCAGTCGACCGGTCCGCGTCGGCCTTCTCGCGCAC  139
PCFVdCaEAs14/981E      CCTTCCTTTCTTCGGGTTTCCTTCCTCAGTCGACCGGTCCGCGTCGGCCTTCTCGCGCAC  139
PCFVdCaAs14/1107E      CCTTCCTTTCTTCGGGTTTCCTTCCTCAGTCGACCGGTCCGCGTCGGCCTTCTCGCGCAC  139
PCFVdCaEAs14/1154E     CCTTCCTTTCTTCGGGTTTCCTTCCTCAGTCGACCGGTCCGCGTCGGCCTTCTCGCGCAC  139
                       ************************************************************

PCFVdCaNK13/1127E      TGCTGTCCGGCTACTACCCGGTGGATACAACTGACAGAGGTGCTTTTTCTTCCACCCGAC  300
PCFVdToNAm13/968E      TGCTGTCCGGCTACTACCCGGTGGATACAACTGACAGAGGTGCTTTTTCTTCCACCCAAC  300
PCFVdCaNK13/1192E      TGCTGTCCGGCTACTACCCGGTGGATACAACTGACAGAGGTGCTTTTTCTTCCACCCGAC  300
KC762952.1             TGCTGTCCGGCTACTACCCGGTGGATACAACTGACAGAGGTGCTTTTTCTTCCACCCAAC  300
KC762953.1             TGCTGTCCGGCTACTACCCGGTGGATACAACTGACAGAGGTGCTTTTTCTTCCACCCGAC  300
PCFVdToAf14/1068E      TGCTGTCCGGCTACT---------------------------------------------  154
PCFVdToME14/0008E      TGCTGTCCGGCTACT---------------------------------------------  154
PCFVdCaME15/707E       TGCTGTCCGGCTACT---------------------------------------------  154
PCFVdToNK13/1170E      TGCTGTCCGGCTACT---------------------------------------------  154
PCFVdToEu13/1252E      TGCTGTCCGGCTACT---------------------------------------------  154
PCFVdCaNAm14/628E      TGCTGTCCGGCTACT---------------------------------------------  154
PCFVdToNAm14/675E      TGCTGTCCGGCTACT---------------------------------------------  154
PCFVdCaEAs14/847E      TGCTGTCCGGCTACT---------------------------------------------  154
PCFVdCaEAs14/981E      TGCTGTCCGGCTACT---------------------------------------------  154
PCFVdCaAs14/1107E      TGCTGTCCGGCTACT---------------------------------------------  154
PCFVdCaEAs14/1154E     TGCTGTCCGGCTACT---------------------------------------------  154
                       ***************                                             

PCFVdCaNK13/1127E      TTCTACCGACGCGGCCGGGAGTGAAGCTACCCGGGACCCGAGAGGATCT    349
PCFVdToNAm13/968E      TTCTACCGACGCGGCCGGGAGTGAAGCTACCCGGGACCCGAGAGGATCT    349
PCFVdCaNK13/1192E      TTCTACCGACGCGGCCGGGAGTGAAGCTACCCGGGACCCGAGAGGATCT    349
KC762952.1             TTCTACCGACGCGGCCGGGAGTGAAGCTACCCGGGACCCGAGAGGATCT    349
KC762953.1             TTCTACCGACGCGGCCGGGAGTGAAGCTACCCGGGACCCGAGAGGATCT    349
PCFVdToAf14/1068E      -------------------------------------------------    154
PCFVdToME14/0008E      -------------------------------------------------    154
PCFVdCaME15/707E       -------------------------------------------------    154
PCFVdToNK13/1170E      -------------------------------------------------    154
PCFVdToEu13/1252E      -------------------------------------------------    154
PCFVdCaNAm14/628E      -------------------------------------------------    154
PCFVdToNAm14/675E      -------------------------------------------------    154
PCFVdCaEAs14/847E      -------------------------------------------------    154
PCFVdCaEAs14/981E      -------------------------------------------------    154
PCFVdCaAs14/1107E      -------------------------------------------------    154
PCFVdCaEAs14/1154E     -------------------------------------------------    154
```

Figure S15. Identical CEVd sequences obtained from different seed lots. CEVdToNK13/042E and CEVdToEu27649 are identical and each has 100% nucleotide identity with Canadian isolate CEVd isolate imp2, (EU877743.1). CEVdToEu13/009E, CEVdToNK13/020E, CEVdToSAm13/008E, CEVdToNK13/45E, CEVdToNK13/019E, CEVdToEu13/046E and CEVdCaEA13/126E are identical and have 99% nucleotide identity with Belgian CEVd isolate GBVdC_CEVd02 (KX084708.1) and Canadian isolate CEVd isolate imp2 (EU877743.1). CEVdToEu13/046E and CEVdCaEA13/126E are identical and have 98% nucleotide identity with Belgian CEVd isolate GBVdC_CEVd02 (KX084708.1).

```
CEVdToEu27649         -----------------------------GCTGCTTCGGTCGCCGCGGATCACTGGCGTC    31
CEVdToEu13/046E       CTGGAGGAAGTCGAGGTCGGGGGGGAACAGCTGCTTCGGTCGCCGCGGATCCCTGGCGTC    60
CEVdCaEA13/126E       CTGGAGGAAGTCGAGGTCGGGGGGGAACAGCTGCTTCGGTCGCCGCGGATCCCTGGCGTC    60
CEVdToNK13/020E       CTGGAGGAAGTCGAGGTCGGGGGGGAACAGCTGCTTCGGTCGCCGCGGATCCCTGGCGTC    60
CEVdToEu13/009E       CTGGAGGAAGTCGAGGTCGGGGGGGAACAGCTGCTTCGGTCGCCGCGGATCCCTGGCGTC    60
CEVdToSAm13/008E      CTGGAGGAAGTCGAGGTCGGGGGGGAACAGCTGCTTCGGTCGCCGCGGATCCCTGGCGTC    60
CEVdToNK13/45E        CTGGAGGAAGTCGAGGTCGGGGGGGAACAGCTGCTTCGGTCGCCGCGGATCCCTGGCGTC    60
CEVdToNK13/019E       CTGGAGGAAGTCGAGGTCGGGGGGGAACAGCTGCTTCGGTCGCCGCGGATCCCTGGCGTC    60
CEVdToNK13/042E       CTGGAGGAAGTCGAGGTCGGGGGGGAACAGCTGCTTCGGTCGCCGCGGATCACTGGCGTC    60
                                                   ********************** ********

CEVdToEu27649         CAGCGGAGAAACAGGAGCTCGTCTCCTTCCTTTCGCTGCTGGCTCCACATCCGATCGTCG    91
CEVdToEu13/046E       CAGCGGAGAAACGGGAGCTCGTCTCCTTCCTTTCGCTGCTGGCTCCACATCCGATCGTCG    120
CEVdCaEA13/126E       CAGCGGAGAAACGGGAGCTCGTCTCCTTCCTTTCGCTGCTGGCTCCACATCCGATCGTCG    120
CEVdToNK13/020E       CAGCGGAGAAACAGGAGCTCGTCTCCTTCCTTTCGCTGCTGGCTCCACATCCGATCGTCG    120
CEVdToEu13/009E       CAGCGGAGAAACAGGAGCTCGTCTCCTTCCTTTCGCTGCTGGCTCCACATCCGATCGTCG    120
CEVdToSAm13/008E      CAGCGGAGAAACAGGAGCTCGTCTCCTTCCTTTCGCTGCTGGCTCCACATCCGATCGTCG    120
CEVdToNK13/45E        CAGCGGAGAAACAGGAGCTCGTCTCCTTCCTTTCGCTGCTGGCTCCACATCCGATCGTCG    120
CEVdToNK13/019E       CAGCGGAGAAACAGGAGCTCGTCTCCTTCCTTTCGCTGCTGGCTCCACATCCGATCGTCG    120
CEVdToNK13/042E       CAGCGGAGAAACAGGAGCTCGTCTCCTTCCTTTCGCTGCTGGCTCCACATCCGATCGTCG    120
                      ************ ***********************************************

CEVdToEu27649         CTGAAGCGCCACGCCCCCTCGCCCGGAGCTTCTCTCTGGAGACTACCCGGTGGAAACAAC    151
CEVdToEu13/046E       CTGAAGCGCCACTCCCCCTCGCCCGGAGCTTCTCTCTGGAGACT----------------    164
CEVdCaEA13/126E       CTGAAGCGCCACTCCCCCTCGCCCGGAGCTTCTCTCTGGAGACT----------------    164
CEVdToNK13/020E       CTGAAGCGCCACGCCCCCTCGCCCGGAGCTTCTCTCTGGAGACT----------------    164
CEVdToEu13/009E       CTGAAGCGCCACGCCCCCTCGCCCGGAGCTTCTCTCTGGAGACT----------------    164
CEVdToSAm13/008E      CTGAAGCGCCACGCCCCCTCGCCCGGAGCTTCTCTCTGGAGACT----------------    164
CEVdToNK13/45E        CTGAAGCGCCACGCCCCCTCGCCCGGAGCTTCTCTCTGGAGACT----------------    164
CEVdToNK13/019E       CTGAAGCGCCACGCCCCCTCGCCCGGAGCTTCTCTCTGGAGACT----------------    164
CEVdToNK13/042E       CTGAAGCGCCACGCCCCCTCGCCCGGAGCTTCTCTCTGGAGACT----------------    164
                      ************ *******************************

CEVdToEu27649         TGAAGCTTCTCTCTGGAGACTACCCGGTGGAAACAACTGAAGC  194
CEVdToEu13/046E       -------------------------------------------  164
CEVdCaEA13/126E       -------------------------------------------  164
CEVdToNK13/020E       -------------------------------------------  164
CEVdToEu13/009E       -------------------------------------------  164
CEVdToSAm13/008E      -------------------------------------------  164
CEVdToNK13/45E        -------------------------------------------  164
CEVdToNK13/019E       -------------------------------------------  164
CEVdToNK13/042E       -------------------------------------------  164
```
