# Supplementary material for: Viroid-infected Tomato and Capsicum Seed Shipments to Australia
Source: Viruses. 2019 Jan 24;11(2):98. doi: 10.3390/v11020098 (PMC6410188; doi:10.3390/v11020098)
Supplement: Supplementary file 1 [file viruses-11-00098-s001.zip › Supplementary file 3 Viruses 415665.pdf]

Supplementary file 3. Viroid sequences.
Isolate Name Convention: Viroid species, host, location, submission ref and first initial of lab
Host: Ca= Capsicum; To = Tomato,
Region: As = Asia; Eu = Europe; EA – east Asia; ME = middle east; Nam = North America; NK = region not known; Sam = South America; Sas – South Asia
Lab: C=CHS; E = EAMI

CLVd

>CLVdCaEu16169C
TTCAGACAGGAGTAATACCAGCTGAAACAGGGTTTTCACCCTTCCTTTCTTCTGGTTTCCTTCCTCTGCTTCAGCGGCCTCG
CCCGGAGTCTTGACCAGCGCAGGTTCTGACGCGACCGGTGGCATCACCGAGTTCGCTCAAGCCTCAACCTCCTTTTTCTTC
ATTCTAGCTTGGTCTCCGGGCGAGGGTGTTTAGCCCTTGGAACCGCAGTTGGTTCCTCGGAACTAAACTCGTGGTTCCTGT
GGTTCACACCTGACCCTGCAGCCATGCAAAGAAAAAAAGAACGGGAGGAAGAGCGCAAGAGCGGTCTCAGGAGCCCCC

>CLVdToEu1413127C
ATAGGAGGGGGTCCTGAGACTGCTAATGCGCTCTTCCTACCGTTCTTTAGTTCTTTGGAAGGTTGCAGGGTCAGGTGTGA
AACACAGGAACCAGGAGTTTAGTTCCGAGGAGCCGTATGCGGTTCCAGGGCTAAACACCCTCGCCCGGACACCATTCTAT
AATGATCAAAAAGGAGGTTGAGGCTTGAGCGAACTCGGTGATGCCGCCGGTCGCGTCAGAACCTGTGCTGGTCAAGACT
CCGGGCGAGGCCGCTGAAGCAGAGGACgGACACcATACTAAAGGAAGGGTGAAAACCCTGTTTCCCCTGGGATTACTCCT
GTCTGAACCGGCCTTTCGCCATTGTTCAAGACCCCGCTCCGTCTGATTTTCCTACTCCCCCC

>CLVdCaEA1302154C
CGGAACTAAACTCGTGGTTCCTGTGGTTCACACCTGACCCTGCAGCCATGCAAAGAAAAAAAGAACGGGAGGAAGAGCG
CAAGAGCGGTCTCAGGAGCCCCTGGGGCAACTCAGACCGAGCGGGGTCTTGACCAGTGGCGAGCGCCCTGTTCAGACAG
GAGTAATCCCAGCTGAAACAGGGTTTTCACCCTTCCTTTCTTCTGGTTTCCTTCCTCTGCTTCAGCGGCCTCGCCCGGAGTC
TTGACCAGCGCAGGTTCTGACGCGACCGGTGGCATCACCGAGTTCGCTCAAGCCTCAACCTCCTTTTTCTTCATTCTAGCTT
GGTCTCCGGGCGAGGGTGTTTAGCCCTTGGAACCGCAGTTGGTTCCT

>CLVdToEu13/684bE (renamed=CLVdToEu1285bE)
CGGAACTAAACTCGTGGTTCCTGTGGTTCACACCTGACCCTGCAGCCATGCAAAGAAAAAAAGAACGGGAGAAAGAGCG
CAAGAGCGGTCTCAGGAGCCCCGGGGCAACTCAGACCGAGCGGGGTCTTGACCAGTGGCGAGCGCCCTGTTCAGACAG
GAGTAATCCCAGCTGAAACAGGGTTTTCACCCTTCCTTTCTTCTGGTTTCCTTCCTCTGCTTCAGCGGCCTCGCCCGGAGTC
TTGACCAGCGCAGGTTCTGACGCGACCGGTGGCATCACCGAGTTTCGCTCAAACCTCAATCTCCTTTTTCTTCATTCTAGCT
TGGTCTCCGGGCGAGGGTGTTTAGCCCTTGGAACCGCAGTTGGTTCCT

>CLVdToME13/159E   (renamed= CLVdToME1155E)
CGGAACTAAACTCGTGGTTCCTGTGGTTCACACCTGACCCTGCGGCCATGCAAAAAAAAAAGAACGGGAGGAAGAGCGC
AAAGAGCGGTCTCAGGAGCCCCGGGGCAACTCAGACCGAGCGGGGTCTTGACCAGTGGCGAGCGCCCTGTTCAGACAG
GAGTAATCCCAGCTGAAACAGGGTTTTCACCCTTCCTTTCTTCTGGTTTCCTTCCTCTGCTTCAGCGGCCTCGCCCGGAGTC
TTGACCAGCGCAGGTTCTGACGCGACCGGTGGCATCACCGAGTTCGCTCAAGCCTCCACCTCCTTTTTCTCTATCTAGCTTG
GTCTCCGGGCGAGGGTGTTTAGCCCTTGGAACCGCAGTTGGTTCCT

>CLVdToME13/421E   (= CLVdToME1273E)
CGGAACTAAACTCGTGGTTCCTGTGGTTCACACCTGACCCTGCAGCCATGCAAAGAAAAAAGAACGGGAGGAAGAGCGC
AAGAGCGGTCTCTGGAGCCCCCCGGGGCAACTCAGACCGAGCGGGGTCTTGACCAGTGGCGAGTGCCCTGTTCAGACAG
GAGTAATCCCAGCTGAAACAGGGTTTTCACCCTTCCTTTCTTCTGGTTTCCTTCCTCTGCTTCAGCGGCCTCGCCCGGAGTC
TTGACCAGCGCAGGTTCTGACGCGACCGGTGGTATCACCGAGTTCGCTCAAGCCTCTACCTCCTTTTTCTCTATCTAGCTTG
GTCTCCGGGCGAGGGTGTTTAGCCCTTGGAACCGCAGTTGGTTCCT

>CLVdToAs13/1059E   (= CLVdToAs1385E)
CGGAACTAAACTCGTGGTTCCTGTGGTTCACACCTGACCCTGCAGCCATGCAAAGAAAAAAAGAACGGGAGGAAGAGCG
CAAGAGCGGTCTCAGGAGCCCCGGGGCAACTCAGACCGAGCGGGGATCGCGGACCGAGGGCGGAAGCCTGCTTCAGAC
AGGAGTAATCCCCGCTGAAACAGGGTTTTCACCCTTCCTTTCTTCGGGTTTCCTTCCTCTGCTTGCGGCCTCGCCCGGAGTC
TTCGAATCCAGCGCAGGTTCTGACGCGACCGGTGGCATCACCGAGTTGCTCAAGCCTCAACCTCCTTTTTCTCTATTCTACG
CTTGGTCTCCGGGCGAGGGTGTTTAGCCCTTGGAACCGCAGTTGGTTCCT

>CLVdToAs13/1073E
CGGAACTAAACTCGTGGTTCCTGTGGTTCACACCTGACCCTGCAGCCATGCAAAGAAAAAAAGAACGGGAGGAAGAGCG
CAAGAGCGGTCTCAGGAGCCCCGGGGCAACTCAGACCGAGCGGGGATCGCGGACCGAGGGCGGAAGCCTGCTTCAGAC
AGGAGTAATCCCCGCTGAAACAGGGTTTTCACCCTTCCTTTCTTCGGGTTTCCTTCCTCTGCTTGCGGCCTCGCCCGGAGTC
TTCGAATCCAGCGCAGGTTCTGACGCGACCGGTGGCATCACCGAGTTGCTCAAGCCTCAACCTCCTTTTTCTCTATTCTACG
CTTGGTCTCCGGGCGAGGGTGTTTAGCCCTTGGAACCGCAGTTGGTTCCT

>CLVdToAs13/1074E
CGGAACTAAACTCGTGGTTCCTGTGGTTCACACCTGACCCTGCAGCCATGCAAAGAAAAAAAGATTGGGAGGAAGAGCG
CAAGAGCGGTCTCAGGAGCCCCGGGGCAACTCAGACCGAGCGGGGATCGCGGACCGAGGGCGGAAGCCTGCTTCAGAC
AGGAGTAATCCCCGCTGAAACAGGGTTTTCACCCTTCCTTTCTTCGGGTTTCCTTCCTCTGCTTGCGGCCTCGCCCGGAGTC
TTCGAATCCAGCGCAGGTTCTGACGCGACCGGTGGCATCACCGAGTTGCTCAAGCCTCATCCTCCTTTTTCTCTATTCTACG
CTTGGTCTCCGGGCGAGGGTGTTTAGCCCTTGGAACCGCAGTTGGTTCCT

>CLVdToAs13/1075E
CGGAACTAAACTCGTGGTTCCTGTGGTTCACACCTGACCCTGCAGCCATGCAAAGAAAAAAGAACGGGAGGAAGAGCGC
AAGAGCGGTCTCAGGAGCCCCGGGGCAACTCAGACCGAGCGGGGATCGCGGACCGAGGGCGGAAGCCTGCTTCAGACA
GGAGTAATCCCCGCTGAAACAGGGTTTTCACCCTTCCTTTCTTCGGGTTTCCTTCCTCTGCTTGCGGCCTCGCCCGGAGTCT
TCGAATCCAGCGCAGGTTCTGACGCGACCGGTGGCATCACCGAGTTGCTCAAGCCTCATCCTCCTTTTTCTCTATTCTACGC
TTGGTCTCCGGGCGAGGGTGTTTAGCCCTTGGAACCGCAGTTGGTTCCT

>CLVdToAs13/1076E
CGGAACTAAACTCGTGGTTCCTGTGGTTCACACCTGACCCTGCAGCCATGCAAAGAAAAAAAGATTGGGAGGGAGAGCG
CAAGAGCGGTCTCAGGAGCCCCGGGGCAACTCAGACCGAGCGGGGATCGCGGACCGAGGGCGGAAGCCTGCTTCTGAC
AGGAGTANNNNNNNNNNNNNNNNNNNNNNNNNNNNNNNNNNNNNNNNTTCCTCTGCTTGCGGCCTCGCCC
GGAGTCTTCGAATCCAGCGCAGGTTCTGACGCGACCGGTGGCATCACCGAGTTGCTCAAGCCTCAACCTCCTTTTTCTCTA
TTCTACGCTTGGTCTCCGGGCGAGGGTGTTTAGCCCTTGGAACCGCAGTTGGTTCCT

>CLVdToAs13/1067E
CGGAACTAAACTCGTGGTTCCTGTGGTTCACACCTGACCCTGCATCCATGCAAAGAAAAAAAGATTGGGAGGAAGAGCG
CAATACCGGTCTCATGAGCCCCGGGGCAACTCAGACCGAGCGGGGATCGCGGACCGAGGGCGGAAGCCTGCTTCTGACT
TGAGTANNNNNNNNNNNNNNNNNNNNNNNNNNNNNNNNNNNNNNNNTTCCTCTGCTTGCGGCCTCGCCCG
GAGTCTTCGAATCCAGCGCAGGTTCTGACGCGACCGGTGGCATCACCGAGTTGCTCAAGCCTCATCCTCCTTTTTCTCTATT
CTACGCTTGGTCTCCGGGCGAGGGTGTTTAGCCCTTGGAACCGCAGTTGGTTCCT

>CLVdToAs13/1065E
CGGAACTAAACTTGTGGTTCCTGTGGTTCACACCTGACCCTGCAGCCATGCAAAGAAAAAAAGATTGGGAGGAAGAGCG
CAAGAGCGGTCTCAGGAGCCCCGGGGCAACTCAGACCGAGCGGGGATCGCGGACCGAGGGCGGAAGCCTGCTTCTGAG
AGGAGTANNNNNNNNNNNNNNNNNNNNNNNNNNNNNNNNNNNNNNNNTTCCTCTGCTTGCGGCCTCGCCC
GGAGTCTTCGAATCCAGCGCAGGTTCTGACGCGACCGGTGGCATCACCGAGTTGCTCAAGCCTCATCCTCCTTTTTCTCTA
TTCTACGCTTGGTCTCCGGGCGAGGGTGTTTAGCCCTTGGAACCGCAGTTGGTTCCT

>CLVdToNK13/1170E
CGGAACTAAACTCGTGGTTCCTGTGGTTCACACCTGACCCTGCAGCCATGCAAAGAAAAAAAGAACGGGAGAAAGAGCG
TAAGAGCGGTCTCAGGAGCCCCGGGGCAACTCAAACCGAGCGGGGTCTTGACCAGGGGCAAGCGCCCTGTTCTGACAGG
AGTAATNNNNNNNNNNNNNNNNNNNNNNNNNNNNNNNNNNNNNNNNTTCCTTTGGTTCAGCGGCCTCGCC
CGGAGTTTTGACCAGCGCAGGTTTTGACGCGACCGGTGGCATCACCGAGTTTCGCTCAAACCTCAAATCTCCTTTTTCTTCA
TTCTAGCTTGGTCTCCGGGCGAGGGTGTTTAGCCCTTGGAACCGCAGTTGGTTCCT

PSTVd

>PSTVdTo1713172C
CTGGAGCGAACTGGCAATAAGGACGGTGGGGAGTGCCCAGCGGCCGACAGGAGTAATTCCCGCCGAAACAGGGTTTTC
ACCCTTCCTTTCTTCGGGTGTCCTTCCTCGCGCCCGCAGGACCACCCCTCGCCCCCTTTGCGCTGTCGCTTCGGCTACT

>PSTVdTo1713158C
CTGGAGCGAACTGGCAATAAGGACGGTGGGGAGTGCCCAGCGGCCGACAGGAGTAATTCCCGCCAAAACAGGGTTTTC
ACCCTTCCTTTTCTGTGGGTGTCCTTCCTCGCGCCCGCAGGTCCACCCCTCGC

>PSTVdCaSAm1302191C
GGGGAAACCTGGAGCGAACTGGCAATAAGGCGGTGGGGAGTGCCCAGCGGCCGACAGGAGTAATTCCCGCCGAAACAG
GGTTTTCACCCTTCCTTTCTTCGGGTGTCCTTCCTCGCGCCCGCAGGACCACCCCTCGCCCCTTTGCGCTGTCGCTTCGGCTA
CTACCCGGTGGAAACAACTGAAGCTCCCGAGAACCGCTTTTTCTCTATCTTCCTTGCTTCGGGGCGAGGGTGTTTAGCCCT
TGGAACCGCAGTTGGTTCCT

>PSTVdToEA130225C
GGGGAAACTTGGAGCGAACTGGCAATAAGGACGGTGGGGAGTGCCCAGCGGCCGACAGGAGTAATTCCCGCCGAAACA
GGGTTTTCACCCTTCCTTTTTTCGGGTGTCCTTCCTCGCGCCCGCAGGACCACCCCTCGCCCCCTTTGAAGCAGTCGGTTCG
GGTAATACCCGGTGGAAACAACTGAAGCTCCCGAGAACCGCTTTTTCTATATCTTACTTTGTTCGGGGCGAGGGTGTTTAG
CCC

>PSTVdToSAm130212C
CGTGGGAAACCTGGAGCGAACTGGCAAAAGGCGCGGTGGGGAGTCCCTCGCGGCCGACAGGAGTAATTCCTGCTGAAA
CAGGGTTTTCACCCTTCCTTTCTTCAGGTTTCCTTCCTCGCGCCCGCAGGATCACCCCTCGCCCCCTTGCGCTGTCGCTTCGG
CTACTACCCGGTGGAAACAACTGAAGCTCCCGAGAACCGCTTTTTCTCTATCTTGCTGGTACCGGGGCGAGGGTGTTTAGC
CCTTGGAACCGCAGTTGGTTCCT

>PSTVdToSAm130211C
AGGGGAAACCTGGAGCCAACTGTCAAAAGGCGCGGTGGGGAGAGCCTCGCGGCCGACAGGAGTAATTCCAGCTGAAAC
AGGGTTTTCACCCTTCCTTTCTTCAGGTTTCCTTCCTCGCGCCCGCAGGATCGCCCCTCGCCCCCTTGCGCTGTCGCTTCGGC
TACTACCCGGTGGAAACAACTGAACCTCCCGAGAACCGCTTTTTCTATATCTTGCTGGTACCGGGTCGAGGGTGTTTAGAC
CTTGGAACCACAGTTGGTTCCT

>PSTVdToEU27651C
CAACAAGGACGGTGGGGAGTGCCCAGCGGCCGACAGGAGTAATTCCCGCCGAAACAGGGTTTTCACCCTTCCTTCCTTCG
GGTGTCCTTCCTCGCGCCCGCAGGACCACCCCTCGCCCCCTTTGCGCTGTCGCTTCGGATACT

>PSTVdToEu27648C
CAAAAGGCGCGGTGGGAAGTGCCTCGCGGCCGACAGGAGTAATTCCTGCCGAAACAGGGTTTTCACCCTTCCTTTCTTCA
GGTTTCCTTCCTCGCGCCCGCAGGATCACCCCTCGCCCCCTTGCGCTGTCGCTTCGGCTACT

>PSTVdToEA27647C
GCGGTGGGGAGTGCCTCGCGACCGACAGGAGTAATTCCTGCCGAAACAGGGTTTTCACCCTTCCTTTCTTCAGGTTTCCTT
CCTCGCGCCCGCAGGATCACCCCTCGCCCCCTTGCGCTGTCGCTTCGGCTACTACCCGGTGGAAACAACT

>PTSVdToME27646C
GGGAGTGCCTCGCGGCCGACAGGAGTAATTCCCGCCGAAACAGGGTTTTCACCCTTCCTTTCTTCGGGTGTCCTTCCTCGC
GCCCGCAGGACCACCCCTCGCCCCCTTTGCGCTGTCGCTTCGGTTACTACCCGGGGGAAACAACTGAAG

>PSTVd1ToME27644C
GTGCCTCGCGGACGACAGGAGTAATTCCTGCCGAAACAGGGTTTTCACCCTTCCTTTCTTCAGGTTTCCTTCCTCGCGCCCG
CAGGATCACCCCTCGCCCCCTTGCGCTGTCGCTTCGGCTACTACCCGGTGGAAACAACTGAAGC

>PSTVd2ToME27644C

CAGTGCCTAGCGGCCGACAGGAGTAATTCCCGCCGAAACAGGGTTTTCACCCTTCCTTTCTTCGGGTGTCCTTCCTCGCGC
CCGCAGGACCACCCCTCGCCCCCTTTGCGCTGTCGCTTCGGTTACTACCCGGTGGAAACAACTGAAGCT

>PSTVdToME27642C
CTGGAGCGAACTGGCAACAAGGACGGTGGGGAGTGCCCAGCGGCCGACAGGAGTAATTCCCGCCGAAACAGGGTTTTC
ACCCTTCCTTTCTTCGGGTGTCCTTCCTCGCGCCCGCAGGACCACCCCTCGCCCCCTTTGCGCTGTCGCTTCGGTTACT

>PSTVdToSAm27638C
GGGATCCCCGGGGAAACCTGGAGCGAACTGGCAATTAGGCGGTGGGGAGTGCCCAACGGCCGACAGGAGTAATTCCCC
CCGAAACAGGGTTTTCACCCTTCCTTTCTTCGGGTGTCCTTCCTCGCGCCCGCAGGACCACCCCTCTC

>PSTVdToSAm27625C
CTGGAGCGAACTGGCAACAAGGACGGTGGGGAGTGCCCAGCGGCCGACAGGAGTAATTCCCGCCGAAACAGGGTTTTC
ACCCTTCCTTTTTTCGGGTGTCCTTCCTCGTGCCCGCAGGACCACCCCTCGCCCCCTTTGCGCTGTCGCTTCGGGTACT

>PSTVdToSAm27610C
CGGGGAAACCTGGAGCGAACTGGCAAAAGGCGCGGTGGGGAGTGCCTCGCGGCCGACAGGAGTAATTCCTGCTGAAAC
AGGGTTTTCACCCTTCCTTTCTTCAGGTTTCCTTCCTCGCGCCCGCAGGATCACCCCTCGCCCCCTTGCGCTGTCGCTTCGGC
TACTACCCGGTGGAAACAACTGAAGCTCCCGAGAACCGCTTTTTCTCTATCTTGCTGGTACCGGGGCGAGGGTGTTTAGCC
CTTGGAACCGCAGTTGGTTCCTC

>PSTVdToSAs27617C
CGGTGGGGAGTGCCCAGCGGCCGACAGGAGTAATTCCCGCCGAAACAGGGTTTTCACCCTTCCTTTCTTCGGGGGTCCAT
CCTCGCGCCCGCAGGACCACCCCTCGCCCCCTTTGCGCTGTCGCTTCGGCTACTACCCGGTGGAAACAACTGAAGCTCCCG
AGAACCGCTTTTTCTCTATCTTACTTTGTTCGGGGCGAGGGTGTTTAGCCCTTGGAACCGCAGTTGGTTCCTCGGAACTAA
ACTCGTGGTTCCTGTGGTTCACACCTGACCTCCTAACAATGCAAAGAAAAAAGAAGGCGGCTCGGAGGAGCGCTTCAGG
A

>PSTVdToMC130219C
GCTTCAGTTGTTTCCCCCGGGTAGTATCCGAAGCGACAGCGCAAGGGGGCGAGGGGTGATCCTGCGGGCGCGAGGAAG
GAAACCTGAAGAAAGGAAGGGTGAAAACCCTGTTTCAGCAGGAATTACTCCTGTCGGCCGCGAGGCACTCCCCACCGCG
CCTTTTGCCAGTTCGCTCCAGGTTTCCCCGGGGATCCACA

>PSTVdToNK12/744E
CTGGAGCGAACTGGCAATAAGGACGGTGGGGAGTGCCCAGCGGCCGACAGGAGTAATTCCCGCCGAAACAGGGTTTTC
ACCCTTCCTTTCTTCGGGTGTCCTTCCTCGCGCCCGCAGGTCCACCCCTCGCCCCCTTTGCGCTGTCGCTTCGGCTAC

>PSTVdToNK12/745E
CTGGAGCGAACTGGCAAAAAAGGACGGTGGGGAGTGCCCAGCGGCCGACAGGAGTAATTCCTGCTGAAACAGGGTTTT
CACCCTTCCTTTCTTCAGGTTTCCTTCCTCGCGCCCGCAGGTCCACCCCTCGCCCCCTTTGCGCTGTCGCTTCGGCTACT

>PSTVdToNK12/820E
CTGGAGCGAACTGGCAAAAGGCGCGGTGGGGAGTGCCTCGCGGCCGACAGGAGTAATTCCTGCCGAAACAGGGTTTTC
ACCCTTCCTTTCTTCAGGTTTCCTTCCTCGCGCCCGCAGGATCACCCCTCGCCCCCTTGCGCTGTCGCTTCGGCTACT

>PSTVdToNK13/020E
CTGGAGCGAACTGGCAAAAAAGGACGGTGGGGAGTGCCCAGCGGCCGACAGGAGTAATTCCTGCTGAAACAGGGTTTT
CACCCTTCCTTTCTTCAGGTTTCCTTCCTCGCGCCCGCAGGTCCACCCCTCGCCCCCTTTGCGCTGTCGCTTCGGCTACT

>PSTVdCaEu13/102E
CTGGAGCGAACTGGCAAAAAAGGACGGTGGGGAGTGCCCAGCGGCCGACAGGAGTAATTCCTGCTGAAACAGGGTTTT
CACCCTTCCTTTCTTCAGGTTTCCTTCCTCGCGCCCGCAGGTCCACCCCTCGCCCCCTTTGCGCTGTCGCTTCGGCTACT

>PSTVdToNK13/567E
CTGGAGCGAACTGGCAAAAAAGGACGGTGGGGAGTGCCCAGCGGCCGACAGGAGTAATTCCTGCTGAAACAGGGTTTT
CACCCTTCCTTTCTTCAGGTTTCCTTCCTCGCGCCCGCAGGTCCACCCCTCGCCCCCTTTGCGCTGTCGCTTCGGCTACT

>PSTVdToEAs13/161E (=PSTVdToEA1291E)
ATCCCCGGGGAAACCTGGAGCGAACTGGCAATAAGGACGGTGGGGAGTGCCCAGCGGCCGACAGGAGTAATTCCCGCC
GAAACAGGGTTTTCACCCTTCCTTTCTTCGGGTGTCCTTCCTCGCGCCCGCAGGACCACCCCTCGCCCCCTTTGCGCTGTCG
CTTCGGCTACTACCCGGTGGAAACAACTGAAGCTCCCGAGAACCGCTTTTTCTCTATCTTTCTTTGCTTCGGGGCGAGGGT
GTTTAGCCCTTGGAACCGCAGTTGGTTCCTCGGAACTAAACTCGTGGTTCCTGTGGTTCACACCTGACCTCCTGAGCAGAA
AAGAAAAAAGAAGGCGGCTCGGAGGAGCGCTTCAGGG

>PSTVdToME13/187E
AGCTTCAGTTGTTTCCACCGGGTAGTAGCCGAAGCGACAGCGCAAGGGGGCGAGGGGTGATCCTGCGGGCGCGAGGAA
GGAAACCTGAAGAAAGGAAGGGTGAAAACCCTGTTTCGGCAGGAATTACTCCTGTCGGCCGCGAGGCACTCCCCACCGC
GCCTTTTGCCAGTTCGCTCCAGGTTTCCCCGGGGATCCC

>PSTVdToME13/818E
CTGGAGCGAACTGGCAAAAGGCGCGGTGGGGAGTGCCTCGCGGCCGACAGGAGTAATTCCTGCCGAAACAGGGTTTTC
ACCCTTCCTTTCTTCAGGTTTCCTTCCTCGCGCCCGCAGGATCACCCCTCGCCCCCTTGCGCTGTCGCTTCGGCTACT

>PSTVdToEAs13/942E
CTGGAGCGAACGGCAATAAGGACGGTGGGGAGTGCCCAGCGGCCGACAGGAGTAATTCCCGCCGAAACAGGGTTTTCA
CCCTTCCTTTCTTCGGGTGTCCTTCCTCGCGCCCGCAGGACCACCCCTCGCCCCCTTTGCGCTGTCGCTTCGGCTACT

>PSTVdTOEu13/967E
CTGGAGCGAACTGGCAAAAGGCGCGGTGGGGAGTGCCTCGCGGCCGACAGGAGTAATTCCTGCCGAAACAGGGTTTTC
ACCCTTCCTTTCTTCAGGTTTCCTTCCTCGCGCCCGCAGGATCCCCCTCGCCCCCTTGCGCTGTCGCTTCGGCTACT

>PSTVdToNK13/1046E
CTGGAGCGAACTGGCAAAAGGCGCGGTGGGGAGTGCCTCGCGGCCGACAGGAGTAATTCCTGCCGAAACAGGGTTTTC
ACCCTTCCTTTCTTCAGGTTTCCTTCCTCGCGCCCGCAGGATCACCCCTCGCCCCCTTGGACTGTCGCTTCGTCTACT

>PSTVdToNK13/1051E
CTGGAGCGAACTGGCAAAAGGCGCGGTGGGGAGTGCCTCGCGGCCGACAGGAGTAATTCCTGCCGAAACAGGGTTTTC
ACCCTTCCTTTCTTCAGGTTTCCTTCCTCGCGCCCGCAGGATCACCCCTCGCCCCCTTGCGCTGTCGCTTCGGCTACT

>PSTVdToAs13/1070E
CTGGAGCGAACTGGCAAAAGGCGCGGTGGGGAGTGCCTCGCGGCCGACAGGAGTAATTCCTGCCGAAACAGGGTTTTC
ACCCTTCCTTTCTTCAGGTTTCCTTCCTCGCGCCCGCAGGATCACCCCTCGCCCCCTTGCGCTGTCGCTTCGGCTACT

>PSTVdToNK13/1100E
CTGGAGCGAACTGGCAAAAGGCGCGGTGGGGAGTGCCTCGCGGCCGACAGGAGTAATTCCTGCCGAAACAGGGTTTTC
ACCCTTCCTTTCTTCAGGTTTCCTTCCTCGCGCCCGCAGGATCACCCCTCGCCCCCTTGCGCTGTCGCTTCGGCTACT

>PSTVdToNK13/1104E
CTGGAGCGAACTGGCAAAAGGCGCGGTGGGGAGTGCCTCGCGGCCGACAGGAGTAATTCCTGCCGAAACAGGGTTTTC
ACCCTTCCTTTCTTCAGGTTTCCTTCCTCGCGCCCGCAGGATCACCCCTCGCCCCCTTGCGCTGTCGCTTCGGCTACT

>PSTVdToNAm13/1206E
CTGGAGCGAACTGGCAAAAGGCGCGGTGGGGAGTGCCTCGCGGCCGACAGGAGTAATTCCTGCCGAAACAGGGTTTTC
ACCCTTCCTTTCTTCAGGTTTCCTTCCTCGCGCCCGCAGGATCACCCCTCGCCCCCTTGCGCTGTCGCTTCGGCTACT

>PSTVdCaNam14/675E
CTGGAGCGAACTGGCAATAAGGACGGTGGGGAGTGCCCAGCGGCCGACAGGAGTAATTCCCGCCGAAACAGGGTTTTC
ACCCTTCCTTTCTTCGGGTGTCCTTCCTCGCGCCCGCAGGTCCACCCCTCGCCCCCTTTGCGCTGTCGCTTCGGCTACT

>PSTVdCaNAm14/627E
CTGGAGCGAACTGGCAATAAGGACGGTGGGGAGTGCCCAGCGGCCGACAGGAGTAATTCCCGCCGAAACAGGGTTTTC
ACCCTTCCTTTCTTCGGGTGTCCTTCCTCGCGCCCGCAGGTCCACCCCTCGCCCCCTTTGCGCTGTCGCTTCGGCTACT
>PSTVdCaNAm14/836E

CTGGAGCGAACTGGCAATAAGGACGGTGGGGAGTGCCCAGCGGCCGACAGGAGTAATTCCCGCCGAAACAGGGTTTTC
ACCCTTCCTTTCTTCGGGTGTCCTTCCTCGCGCCCGCAGGACCACCCCTCGCCCCCTTTGCGCTGTCGCTTCGGCTACT

>PSTVdCaNAm15/1046E
CTGGAGCGAACTGGCAATAAGGACGGTGGGGAGTGCCCAGCGGCCGACAGGAGTAATTCCCGCCGAAACAGGGTTTTC
ACCCTTCCTTTCTTCGGGTGTCCTTCCTCGCGCCCGCAGGTCCACCCCTCGCCCCCTTTGCGCTGTCGCTTCGGCTACT

>PSTVdToNK12/868E
CTGGAGTGAACTGGCAAAAAAGGACGGTGGGGAGTGCCCAGCGGCCGACAGGAGTAATTCCTGCTGAAACAGGGTTTT
CACCCTTCCTTTCTTCAGGTTTCCTTCCTCGCGCCCGCAGGTCCACCCCTCGCCCCCTTTGCGCTGTCGCTTCGGCTACT

>PSTVdToEu13/101bE
GGGTAGTGCCCAGCGGCCGACAGGAGTAATTCCTGCTGAAACAGGGTTTTCACCCTTCCTTTCTTCAGGTTTCCTTCCTCG
CGCCCGCAGGTCCACCCCTCGCCCCCTTTGCGCTGTCGCTTCGGCTACTACCCGGTGGATACAACTGAAGCT

>PSTVdCaAf13/124E
CTGGAGCGAACTGGCAATAAGGACGGTGGGGAGTGCCCAGCGGCCGACAGGAGTAATTCCCGCCGAAACAGGGTTTTC
ACCCTTCCTTTCTTCGGGTGTCCTTCCTCGCGCCCGCAGGACCACCCCTCGCCCCCTTTGCGCTGTCGCTTCGGCTACT

>PSTVdToNK13/1205E
CTGGAGCGATCTGGCAAAAAAGGGCGGTGGGGAGCGGCCAGCGGCCGACAGGAGTAATTCGTGCTGAAACAGGGTTTT
CACCCTTCCTTTCTTCAAGTTTCCTTCCTCACGCCCGCAGGTCCACCCCTCGCCCCCTTTGCGCTGTCGCTTCGGCTACT

PCFVd

>KC762953.1 Pepper chat fruit viroid isolate EMAI12/759, complete genome
CCGGATTCTTCTAAGGGTGCCTGTGGTGCCTCCCCCGAAGCCCGCTTAGGGAAAAAGAAAGGGGAAGCAA
GCATCTCCTGTTCAGGGATCCCCGGGGAAACCTGGACAGACCGGGCGGAGAAGCGCACGAGCGGGACCGT
CTTCTGACAGGAGTAATCCCCGCTGAAACAGGGTTTTCACCCTTCCTTTCTTCGGGTTTCCTTCCTCAGT
CGACCGGTCCGCGTCGGCCTTCTCGCGCACTGCTGTCCGGCTACTACCCGGTGGATACAACTGACAGAGG
TGCTTTTTCTTCCACCCGACTTCTACCGACGCGGCCGGGAGTGAAGCTACCCGGGACCCGAGAGGATCT

>KC762954.1 Pepper chat fruit viroid isolate EMAI12/761, complete genome
CCGGATTCTTCTAAGGGTGCCTGTGGTGCCTCCCCCGAAGCCCGCTTAGGGAAAAAGAAAGGGGAAGCAA
GCATCTCCTGTTCAGGGATCCCCGGGGAAACCTGGACAGACCGGGCGGATAAGCGCACGAGCGGGACCGT
CTTCTGACAGGAGTAATCCCCGCTGAAACAGGGTTTTCACCCTTCCTTTCTTCGGGTTTCCTTCCTCAGT
CGACCGGTCCGCGTCGGCCTTCTCGCGCACTGCTGTCCGGCTACTACCCGGTGGATACAACTGACAGAGG
TGCTTTTTCTTCCACCCGACTTCTACCGACGCGGCCGGGAGTGAAGCTACCCGGGACCCGAGAGGATCT

>KC762952.1 Pepper chat fruit viroid isolate EMAI12/80420, complete genome
CCGGATTCTTCTAAGGGTGCCTGTGGTGCCTCCCCCGAAGCCCGCCTAGGGAAAAAGAAAGGGGAAGCAA
GCATCTCCTGTTCAGGGATCCCCGGGGAAACCTGGACAGACCGGGCGGAGAAGCGCACGAGCGGGACCGT
CTTCTGACAGGAGTAATCCCCGCTGAAACAGGGTTTTCACCCTTCCTTTCTTCGGGTTTCCTTCCTCAGT
CGACCGGTCCGCGTCGGCCTTCTCGCGCACTGCTGTCCGGCTACTACCCGGTGGATACAACTGACAGAGG
TGCTTTTTCTTCCACCCAACTTCTACCGACGCGGCCGGGAGTGAAGCTACCCGGGACCCGAGAGGATCT

>PCFVdToME13/159E
CCGGATTCTTCTAAGGGTGCCTGTGGTGCCTCCCCCGAAGCCCGCTTAGGGAAAAAGAAAGGGGAAGCAAGCATCTCCT
GTTCAGGGATCCCCGGGGAAACCTGGACAGACCGGGCGGAGAAGCGCACGAGCGGGACCGTCTTCTGACAGGAGTAAT
CCCCGCTGAAACAGGGTTTTCACCCTTCCTTTCTTCGGGTTTCCTTCCTCAGTTGACCGGTCCGCGTCGGCCTTCTCGCGCA
CTGCTGTCCGGCTACTACCCGGTGGATACAACTGACAGAGGTGCTTTTTCTTCCACCCGACTTCTACCGACGCGGCCGGGA
GTGAAGCTACCCGGGACCCGAGAGGATCT

>PCFVdToNAm13/968E
CCGGATTCTTCTAAGGGTGCCTGTGGTGCCTCCCCCGAAGCCCGCTTAGGGAAAAAGAAAGGGGAAGCAAGCATCTCCT
GTTCAGGGATCCCCGGGGAAACCTGGACAGACCGGGCGGAGAAGCGCACGAGCGGGACCGTCTTCTGACAGGAGTAAT

CCCCGCTGAAACAGGGATTTCTCCCTTCCTTTCTTCGGGTTTCCTTCCTCAGTCGACCGGTCCGCGTCGGCCTTCTCGCGCA
CTGCTGTCCGGCTACTACCCGGTGGATACAACTGACAGAGGTGCTTTTTCTTCCACCCAACTTCTACCGACGCGGCCGGGA
GTGAAGCTACCCGGGACCCGAGAGGATCT

>PCFVdToAs13/1072E
CCGGATTCTTCTAAGGGTGCCTGTGGTGCCTCCCCCGAAGCCCGCTTAGGGAAAAAGAAAGGGGAAGCAAGCATCTCCT
GTTCAGGGATCCCCGGGGAAACCTGAACAGATCGGGCGGAAAAGCGCCGTGCGGGTCCGTCTTCTGACAGGACTANNN
NNNNNNN**NNNNNNNNNNNNNNNNNNNNNNNNNNNNNNNNNNNN**TTCCTCAGTCGACCGGACCGCGTCGGCCTT
CTCGCGCACTTCTGTTCGGAGACTACCCGGTGGATACAACTGACAGAGGTGCTTTTTCTTCCACCCTGCTTCTACCGACGC
GGCCGGGAGTGAAGCTACCCGGGACCCGAGAGGATCT

>PCFVdCaNK13/1127E
CCGGATTCTTCTAAGGGTGCCTGTGGTGCCTCCCCCGAAGCCCGCTTAGGGAAAAAGAAAGGGGAAGCAAGCATCTCCT
GTTCAGGGATCCCCGGGGAAACCTGGACAGACCGGGCGGAGAAGCGCACGAGCGGGACCGTCTTCTGACAGGAGTAAT
CCCCGCTGAAACAGGGATTTTTCCCTTCCTTTCTTCGGGTTTCCTTCCTCAGTCGACCGGTCCGCGTCGGCCTTCTCGCGCA
CTGCTGTCCGGCTACTACCCGGTGGATACAACTGACAGAGGTGCTTTTTCTTCCACCCGACTTCTACCGACGCGGCCGGGA
GTGAAGCTACCCGGGACCCGAGAGGATCT

>PCFVdCaNK13/1192E
CCGGATTCTTCTAAGGGTGCCTGTGGTGCCTCCCCCGAAGCCCGCTTAGGGAAAAAGAAAGGGGAAGCAAGCATCTCCT
GTTCAGGGATCCCCGGGGAAACCTGGACAGACCGGGCGGAGAAGCGCACGAGCGGGACCGTCTTCTGACAGGAGTAAT
CCCCGCTGAAACAGGGTTTTCACCCTTCCTTTCTTCGGGTTTCCTTCCTCAGTCGACCGGTCCGCGTCGGCCTTCTCGCGCA
CTGCTGTCCGGCTACTACCCGGTGGATACAACTGACAGAGGTGCTTTTTCTTCCACCCGACTTCTACCGACGCGGCCGGGA
GTGAAGCTACCCGGGACCCGAGAGGATCT

>PCFVdToNK13/1170E
CTGGACAGACCGGGCGGAGAAGCGCACGAGCGGGACCGTCTTCTGACAGGAGTAATCCCCGCTGAAACAGGGTTTTCAC
CCTTCCTTTCTTCGGGTTTCCTTCCTCAGTCGACCGGTCCGCGTCGGCCTTCTCGCGCACTGCTGTCCGGCTACT

>PCFVdToEu13/1252E
CTGGACAGACCGGGCGGAGAAGCGCACGAGCGGGACCGTCTTCTGACAGGAGTAATCCCCGCTGAAACAGGGTTTTCAC
CCTTCCTTTCTTCGGGTTTCCTTCCTCAGTCGACCGGTCCGCGTCGGCCTTCTCGCGCACTGCTGTCCGGCTACT

>PCFVdCaEAs13/1317E
CGGAACTAAACTCGTGGTTCCTGTGGTTCACACCTGACCCTGCAGCCATGCAAAGAAAAAAGAACGGGAGGAAGAGCGC
GAAGAGCGGTCTCAGGAGCCCCGGGGCAACTCAGACCGAGCGGGGTCTTGACCAGTGGCGAGCGCCCTGTTCAGACAG
GAGTAATCCCGGCTGAAACAGGGTTTTCACCCTTCCTTTCTTCCGGTTTCCTTCCTCTGCTTCAGCGGCCTCGCCCGGAGTC
TTGACCAGCGCAGGTTCTGACGCGACCGGTGGCATCACCGAGTTCGCTCAAGCCTCAACCTCCTTTTTCTCTATCTAGCTTG
GTCTCCGGGCGAGGGTGTTTAGCCCTTGGAACCGCAGTTGGTTCCT

>PCFVdToME14/0008E
CTGGACAGACCGGGCGGAGAAGCGCACGAGCGGGACCGTCTTCTGACAGGAGTAATCCCCGCTGAAACAGGGATTTCTC
CCTTCCTTTCTTCGGGTTTCCTTCCTCAGTCGACCGGTCCGCGTCGGCCTTCTCGCGCACTGCTGTCCGGCTACT

>PCFVdToNAm14/601E
CTGGACAGACCGGGCGGAGAAGCGCACGAGCGGGACCGTCTTCTGACAGGAGTAATCCCCGCTGAAACAGGGTTTTCAC
CCTTCCTTTCTTCGGGTTTCCTTCCTCAGTCAACCGGTCCGCGTCGGCCTTCTCGCGCACTGCTGTCCGGCTACT

>PCFVdCaNAm14/628E
CTGGACAGACCGGGCGGAGAAGCGCACGAGCGGGACCGTCTTCTGACAGGAGTAATCCCCGCTGAAACAGGGTTTTCAC
CCTTCCTTTCTTCGGGTTTCCTTCCTCAGTCGACCGGTCCGCGTCGGCCTTCTCGCGCACTGCTGTCCGGCTACT

>PCFVdToNAm14/675E
CTGGACAGACCGGGCGGAGAAGCGCACGAGCGGGACCGTCTTCTGACAGGAGTAATCCCCGCTGAAACAGGGTTTTCAC
CCTTCCTTTCTTCGGGTTTCCTTCCTCAGTCGACCGGTCCGCGTCGGCCTTCTCGCGCACTGCTGTCCGGCTACT

>PCFVdToNAm14/677E
CTGGACAGACCGGGCGGAGAAGCGCACGAGCGGGACCGTCTTCTGACAGGAGTAATCCCCGCTGAAACAGGGATTTCTC
CCTTCCTTTCTTCGGGTTTCCTTCCTCAGTCAACCGGTCCGCGTCGGCCTTCTCGCGCACTGCTGTCCGGCTACT

>PCFVdCaEAs14/847E
CTGGACAGACCGGGCGGAGAAGCGCACGAGCGGGACCGTCTTCTGACAGGAGTAATCCCCGCTGAAACAGGGTTTTCAC
CCTTCCTTTCTTCGGGTTTCCTTCCTCAGTCGACCGGTCCGCGTCGGCCTTCTCGCGCACTGCTGTCCGGCTACT

>PCFVdCaEAs14/981E
CTGGACAGACCGGGCGGAGAAGCGCACGAGCGGGACCGTCTTCTGACAGGAGTAATCCCCGCTGAAACAGGGTTTTCAC
CCTTCCTTTCTTCGGGTTTCCTTCCTCAGTCGACCGGTCCGCGTCGGCCTTCTCGCGCACTGCTGTCCGGCTACT

>PCFVdToAf14/1068E
CTGGACAGACCGGGCGGAGAAGCGCACGAGCGGGACCGTCTTCTGACAGGAGTAATCCCCGCTGAAACAGGGATTTCAC
CCTTCCTTTCTTCGGGTTTCCTTCCTCAGTCGACCGGTCCGCGTCGGCCTTCTCGCGCACTGCTGTCCGGCTACT

>PCFVdCaAs14/1107E
CTGGACAGACCGGGCGGAGAAGCGCACGAGCGGGACCGTCTTCTGACAGGAGTAATCCCCGCTGAAACAGGGTTTTCAC
CCTTCCTTTCTTCGGGTTTCCTTCCTCAGTCGACCGGTCCGCGTCGGCCTTCTCGCGCACTGCTGTCCGGCTACT

>PCFVdCaEAs14/1154E
CTGGACAGACCGGGCGGAGAAGCGCACGAGCGGGACCGTCTTCTGACAGGAGTAATCCCCGCTGAAACAGGGTTTTCAC
CCTTCCTTTCTTCGGGTTTCCTTCCTCAGTCGACCGGTCCGCGTCGGCCTTCTCGCGCACTGCTGTCCGGCTACT

>PCFVdCaNAm14/1153E
CTGAACAGATCGGGCGGAGAAGCGCCGTGCGGGTCCGTCTTCTGACAGGAGTAATCCCCGCTGAAACAGGGTTTTCACC
CTTCCTTTCTTCGGGTTTCCTTCCTCAGGCGACCGGACCGCGTCGGCCTTCTCGCGCACTTCTGTTCGGAGACT

>PCFVdCaME15/707E
CTGGACAGACCGGGCGGAGAAGCGCACGAGCGGGACCGTCTTCTGACAGGAGTAATCCCCGCTGAAACAGGGATTTCTC
CCTTCCTTTCTTCGGGTTTCCTTCCTCAGTCGACCGGTCCGCGTCGGCCTTCTCGCGCACTGCTGTCCGGCTACT

>PCFVdCaEAs15/913E
CTGAACAGATCGGGCGGAGAAGCGCCGTGCGGGTCCGTCTTCTGACAGGAGTAATCCCCGCTGAAACAGGGTTTTCACC
CTTCCTTTCTTCGGGTTTCCTTCCTCAGTCGACCGGACCGCGTCGGCCTTCTCGCGCACTTCTGTTCGGAGACT

>PCFVdCaNAm15/1046E
CTGGAGCGAACTGGCAATAAGGACGGTGGGGAGTGCCCAGCGGCCGACAGGAGTAATTCCCGCCGAAACAGGGTTTTC
ACCCTTCCTTTCTTCGGGTGTCCTTCCTCGCGCCCGCAGGTCCACCCCTCGCCCCCTTTGCGCTGTCGCTTCGGCTACT

>PCFVdCaAs16/56E
CCGGATTCTTCTAAGGGTGCCTGTGGTGCCTCCCCCGAAGCCCGCTTAGGGAAAAAGAAAGGGGAAGCAAGCATCTCCT
GTTCAGGGATCCCCGGGGAAACCTGGACAGACCGGGCGGAGAAGCGCACGAGCGGGACCGTCTTCTGACAGGAGTAAT
NNNNNNNNNNNNNNNNNNNNNNNNNNNNNNNNNNNNNNNNNNNNNNNNNNNTTCCTCAGTCGACCGGTCCGCGTCGGC
CTTCTCGCGCACTGCTGTCCGGCTACTACCCGGTGGATACAACTGACAGAGGTGCTTTTTCTTCCACCCGACTTCTACCGAC
GCGGCCGGGAGTGAAGCTACCCGGGACCCGAGAGGATCT

CEVd

>CEVdToEu27649
GCTGCTTCGGTCGCCGCGGATCACTGGCGTCCAGCGGAGAAACAGGAGCTCGTCTCCTTCCTTTCGCTGCTGGCTCCACAT
CCGATCGTCGCTGAAGCGCCACGCCCCCTCGCCCGGAGCTTCTCTCTGGAGACTACCCGGTGGAAACAACTGAAGCTTCT
CTCTGGAGACTACCCGGTGGAAACAACTGAAGC

>CEVdToEu13/009E
CTGGAGGAAGTCGAGGTCGGGGGGGAACAGCTGCTTCGGTCGCCGCGGATCCCTGGCGTCCAGCGGAGAAACAGGAGC
TCGTCTCCTTCCTTTCGCTGCTGGCTCCACATCCGATCGTCGCTGAAGCGCCACGCCCCCTCGCCCGGAGCTTCTCTCTGGA
GACT

>CEVdToNK**13/020**E
CTGGAGGAAGTCGAGGTCGGGGGGGAACAGCTGCTTCGGTCGCCGCGGATCCCTGGCGTCCAGCGGAGAAACAGGAGC
TCGTCTCCTTCCTTTCGCTGCTGGCTCCACATCCGATCGTCGCTGAAGCGCCACGCCCCCTCGCCCGGAGCTTCTCTCTGGA
GACT

>CEVdToSAm13/008E
CTGGAGGAAGTCGAGGTCGGGGGGGAACAGCTGCTTCGGTCGCCGCGGATCCCTGGCGTCCAGCGGAGAAACAGGAGC
TCGTCTCCTTCCTTTCGCTGCTGGCTCCACATCCGATCGTCGCTGAAGCGCCACGCCCCCTCGCCCGGAGCTTCTCTCTGGA
GACT

>CEVdToNK13/042E
CTGGAGGAAGTCGAGGTCGGGGGGGAACAGCTGCTTCGGTCGCCGCGGATCACTGGCGTCCAGCGGAGAAACAGGAGC
TCGTCTCCTTCCTTTCGCTGCTGGCTCCACATCCGATCGTCGCTGAAGCGCCACGCCCCCTCGCCCGGAGCTTCTCTCTGGA
GACT

>CEVdToNK13/45E
CTGGAGGAAGTCGAGGTCGGGGGGGAACAGCTGCTTCGGTCGCCGCGGATCCCTGGCGTCCAGCGGAGAAACAGGAGC
TCGTCTCCTTCCTTTCGCTGCTGGCTCCACATCCGATCGTCGCTGAAGCGCCACGCCCCCTCGCCCGGAGCTTCTCTCTGGA
GACT

>CEVdCaEu13/74Be
CTGGAGGAAGTCGAGGTCGGGGGGGAACAGCTGCTTCGGTCGCCGCGGATCACTGGCGTCCAGCGGAGAAACAGGAGC
TCGTCTCCTTCCTTTCGCTGCTGGCTCCACATCCGATCGTCGCTGAAGCGCCACGCCCCCTCGCCCGGAGCTTCTCTCTGGC
TACT

>CEVdToNK13/019E
CTGGAGGAAGTCGAGGTCGGGGGGGAACAGCTGCTTCGGTCGCCGCGGATCCCTGGCGTCCAGCGGAGAAACAGGAGC
TCGTCTCCTTCCTTTCGCTGCTGGCTCCACATCCGATCGTCGCTGAAGCGCCACGCCCCCTCGCCCGGAGCTTCTCTCTGGA
GACT

>CEVdToEu13/044E
CTGGAGGAAGTCGAGGTCGGGGGGGAACAGCTGATTCGGTCGCCGCGGATCCCTGGCGTCCAGCGGAGAAACAGGAGC
TCGTCTCCTTCCTTTCGCTGCTGGCTCCACATCCGATCGTCGCTGAAGCGCCACGCCCCCTCGCCCGGAGCTTCTCTCTGGA
GACT

>CEVdToEu13/046E
CTGGAGGAAGTCGAGGTCGGGGGGGAACAGCTGCTTCGGTCGCCGCGGATCCCTGGCGTCCAGCGGAGAAACGGGAGC
TCGTCTCCTTCCTTTCGCTGCTGGCTCCACATCCGATCGTCGCTGAAGCGCCACTCCCCCTCGCCCGGAGCTTCTCTCTGGA
GACT

>CEVdCaEu13/102E
CTGGAGGAAGTCGAGGTCGGGGGGGAACAGCTGCTTCGGTCGCCGCGGATCCCTGGCGTCCAGCGGAGAAACAGGAGC
TCGTCTCCTTCCTTTCGCTGCTGGCTCCACATCCGATCGTCGCTGAAGCGCCACGCCCCCTCGCCCGGAGCTTCTCTCTGGA
GACT

>CEVdCaEu13/139E
CTGGAGGAAGTCGAGGTCGGGGGGGAACAGCTGCTTCGGTCGCCGCGGATCACTGGCGTCCAGCGGAGAAACAGGAGC
TCGTCTCCTTCCTTTCGCTGCTGGCTCCACATCCGATCGTCGCTGAAGCGCCACGCCCCCTCGCCCGGAGCTTCTCTCTGGA
GACT

>CEVdCaEA13/126E
CTGGAGGAAGTCGAGGTCGGGGGGGAACAGCTGCTTCGGTCGCCGCGGATCCCTGGCGTCCAGCGGAGAAACGGGAGC
TCGTCTCCTTCCTTTCGCTGCTGGCTCCACATCCGATCGTCGCTGAAGCGCCACTCCCCCTCGCCCGGAGCTTCTCTCTGGA
GACT

>CEVdCaAf13/456E
CAGGAGGAAGTCGAGGTCGGGGGGGAACAGCTGCTTCGGTCGCCGCGGATCCCTGGCGTCCAGCGGAGAAACAGGAGC
TCGTCTCCTTCCTTTCTCTGCTGGCTCCTCATCCGATCGTCGCTGAAGCGCCACGCCCCCTCGCCCGGAGCTTCTCTCTGGA
GACT

>CEVdToNK13/1152E
CTGGAGGAAGTCGAGGACGGGGGCTCGGAAGATGCGGTCGCCGCGGATCACTGGCGTCCAGCGGAGAAACAGGAGCT
CGACTCCTTCCTTTCGCTGCTGGCTCCACATCCGATCGTCGCTGCAGTCTCTTGACCCCTCGCCCGGAGCTTCTCTCTGGCT
ACT

TASVd

>TASVdToAs13/1072E (= TASVdToAs1433E)
CATCAAGAAAAAAGATAGGCGCGGGAGCGAAGAAGTCCTTCAGGGATCCCCGGGGAAACCCGAAGGAAGTCGAGGAC
GGGGGCTTCCGACTACTCCCGTGTGAGACAGGAGTAATCCCAGCTGAAACAGGGTTTTCACCCTTCCTTTCTTCTGGTTTC
CTTCCTCTCGCCTGGAGAGGTCTTCGGCCCTCGCCCGGAGCTTCTCTTAGGAGACTACCCGGTGGATACAACTGAAGCTTC
AACCTCCACGCGCTTTTTCTCTATCTTTGTTGCTCTCCGGGCGAGGGTGAAAGCCCGTGGAACCCTACTAAGAGTCCCT

> TASVdToAs13/1073E (= TASVdToNK1435E)
CATCAAGAAAAAAGATAGGCGCGGGAGCGAAGAAGTCCTTCAGGGATCCCCGGGGAAACCCGAAGGAAGTCGAGGAC
GGGGGCTTCCGACTACTCCCGTGTGAGACAGGAGTAATCCCAGCTGAAACAGGGTTTTCACCCTTCCTTTCTTCTGGTTTC
CTTCCTCTCGCCTGGAGAGGTCTTCGGCCCTCGCCCGGAGCTTCTCTTAGGAGACTACCCGGTGGATACAACTGAAGCTTC
AACCTCCTCGCGCTTTTTCTCTATCTTTGTTGCTCTCCGGGCGAGGGTGAAAGCCCGTGGAACCCTAGTAAGAGTCCCT

TCDVd

>TCDVdToAf130222C
GAACAGGAGTAATCCCGAGAGAAACAGGGTTTTCACCCTTCCTTTCTGATTCGGTTTCCTTCCTTTGCGCGCCACTCGACCC
CTCGCCCCCTTGCGCTGTCGCTTCGGCAACTACCCGGTGGAAACAACTGAAGCTCCCGAGAACCGCTTTTTCTCTATCTTGC
TGCTACCGGGGCGAGGGTGTTTAGCCCTTGGAACCGCAGTTGGTTCCTCGGAACTAAACTCGTGGTTCCTGTGGTTCAGA
CCTGACCTCCTGTGCAGAAAAGAAAAAAGATAGGCGGCTCGGAGGAGCGCTTCAGGA

> TCDVdToAf130227C
GGGGAAACCTGGAGCGAACTGGCAAAAGGCGGCAGGGAGCTTGTGGAAGGCGAAACAGGAGTAATCCCGAGAGAAAC
AGGGTTTTCACCCTTCCTTTCTGATTCGGTTTCCTTCCTTTGCGCGCCACTCGACCCCTCGCCCCCTTGCGCTGTCGCTTCGG
CAACTACCCGGTGGAAACAACTGAAGCTCCCGAGAACCGCTTTTTCTCTATCTTGCTGCTACCGGGGCGAGGGTGTTTAGC
CCTTGGAACCGCAGTTGGTTCCTCGGAACTAAACTCGTGGTTCCTGTGGTTCACACCTGACCTCCTGTGCAGAAAAGAAAA
AAGATAGGCGGCTCGGAGGAGCGCTTCAGGATAGGCCATGT

>TCDVdTAf130221C
AGGCGAAACAGGAGTAATCCCGAGAGAAAcAGGGATTTCACCCTTCCTTTCTGATTCGGTTTCCTTCCTTTGCGTGCCACT
CGACCCCTCGCCCCCTTGCGCTGTCGCTTCGGGAACTACCCGGTGGAAACAACTGAAGCTCCCGAGAACCGCTTTTTCTCT
ATCTTGATGCTACCGGGGCGAGGGTGTTTAGCCCTTGGAACCGCAGTTGGTTCCTCGGAACTAAACTCGTGTCTCCTGTG
GTTCAGACCTGACCTCCTGTGCATAAAAGAAAAAAGATAGGCGGCTCG

>TCDVdToSAm130225C
GGCGAGGGGTCGAGTGGCGCGCAAAGGAAGGAAACCGAATCAGAAAGGAAGGGTGAAAACCCTGTTTCTCTCGGGATT
ACTCCTGTTTCGCCTTCCACAAGCTCCCTGCCGCCTTTTGCCAGTTCGCTCCAGGTTTCCCCGGGGATCCC

>TCDVdToEuMC27648C
GCTTGTGGAGGCGAaACAGGAGTAATCCCGAGAGAAACAGGGTTTTCACCCTTCCTTTCTGATTCGGTTTCCTTCCTTTGC
GCGCCACTCGACCCCTCGCCCCCTTGCGCTGTCGCTTCGGCAACTACCCGGGGGAAACAACTGAAGC

>TCDVdToMEMC27642C
ATCCCCGGGGAAACCTGGAGCGAACTGGCAAAAGGCGGCAGGGAGCTTGTGGAAGGCGAAACAGGAGTAATCCCGAG
AGAAACAGGGTTTTCACCCTTCCTTTCTGATTCGGTTTCCTTCCTTTGCGCGCCACTCGACCCCTCGCCCCCTTGCGCTGTCG
CTTCGGCAACTACCCGGTGGAAACAACTGAAGCTCCCGAGAACCGCTTTTTCTCTATCTTGCTGCTACCGGGGCGAGGGT
GTTTAGCCCTTGGAACCGCAGTTGGTTCCTCGGAACTAAACTCGTGGTTCCTGTGGTTCACACCTGACCTCCTGTGCAGAA
AAGAAAAAAGATAGGCGGCTCGGAGGAGCGCTTCAGG

>TCDVdToEu276_34C
TCAGGGATTCCCGGGGAAACCTGGAGCGAACTGGCAAAAGGCGGCAGGGAGCTGGTGGAAGGCGAAACAGGAGTAAT
CCCGAGAGAAACAGGGTTTTCACCCTTCCTTTCTGATTCGGTTTCCTTCCTTTGCGCGCCACTCGACCCCTCGCCCCCTTGC
GCGGTGGTTGCGGTAACTACCCGGTGGAAACAACTGAAGCT

>TCDVdToEu27618C
CTTGTGGAAGGCGAAACAGGAGTAATCCCGAGAGAAACAGGGTTTTCACCCTTCCTTTCTGATTCGGTTTCCTTCCTTTGC
GCGCCACTCGACCCCTCGCCCCCTTGCGGTGACGGTTCGGCAACTACCCGGTGGAAACAACTGAAGCTCCCGAGAACCGC
TTTTTCTCTATCTTGCTGCTACCGGGGCGAGGGTGTTTAGCCCTTGGAACCGCAGTTGGTTCCTCGGAACTAAACTCGTGG
TTCCTGTGGTTCACACCTGACCTCCTGTGCAGAAAAGAAAAAAGATAGGCGGCTCGGAGGAGCGCTTCAGG

>TCDVdToNam13593C
ATAACATGGGGATCCCCGGGGAAACCTGGAGCGAACTGGCAAAAGGCGGCAGGGAGCTTGTGGAAGGCGAAACAGGA
GTAAGCCCGCGAGAAACAGGGTTTTCACCCTTCCTTTCTGACGCGGCTTCCTTCCTTTGCGCGCCACTCGACCCCTCGCCCC
CTTGCGCTGTCGCTTCGGCTACTACCCGGGGGATACAACTGAAGCAAACAGTTGTTA
